# Thermally Reversible Organocatalyst for the Accelerated Reprocessing of Dynamic Networks with Creep Resistance

**DOI:** 10.1021/acsmacrolett.3c00544

**Published:** 2023-11-01

**Authors:** Giulia Vozzolo, Marta Ximenis, Daniele Mantione, Mercedes Fernández, Haritz Sardon

**Affiliations:** † POLYMAT, University of the Basque Country UPV/EHU, Joxe Mari Korta Center, Avda. Tolosa 72, 20018 Donostia-San Sebastian, Spain; ‡ Ikerbasque, Basque Foundation for Science, 48013 Bilbao, Spain

## Abstract

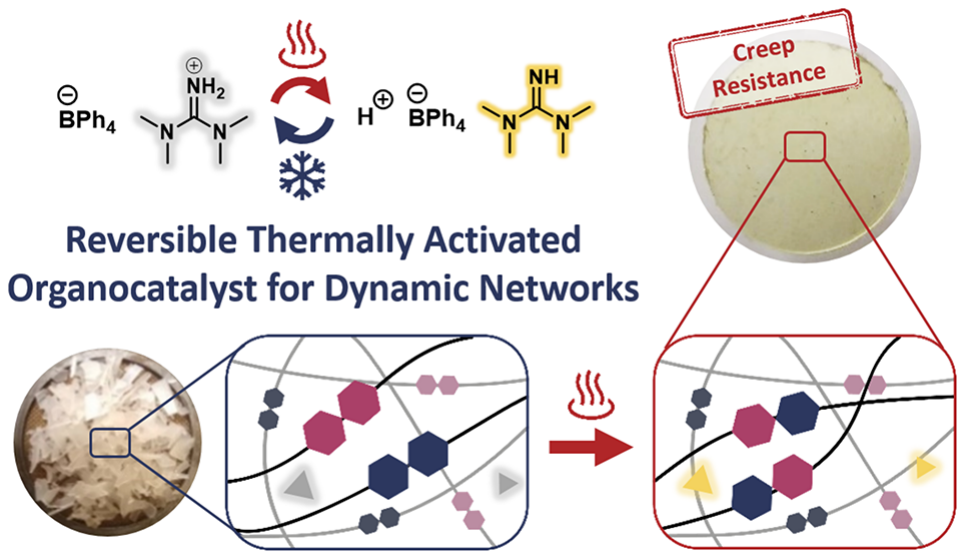

The industrial implementation of covalent adaptable networks hinges on the delicate task of achieving rapid bond exchange activation at specific temperatures while ensuring a sufficiently slow exchange at working temperatures to avoid irreversible deformation. In this pursuit, latent catalysts offer a potential solution, allowing for spatiotemporal control of dynamic exchange in vitrimer networks. However, the irreversible nature of their activation has led to undesired creep deformation after multiple cycles of reprocessing. In this work, we demonstrate that a tetraphenylborate tetramethyl guanidinium salt (**TPB:TMG**) undergoes a reversible thermal dissociation, releasing free **TMG**. This thermally reversible organocatalyst can be readily introduced as an additive in industrially relevant materials such as disulfide-containing polyurethane networks (PU) that undergo disulfide exchange in the presence of a base catalyst. In contrast with a free-base-catalyzed process, we demonstrate the dual benefit of adding the thermally reversible **TPB:TMG** in preventing creep at lower temperatures and also enabling reprocessability of disulfide-containing PU networks at elevated temperatures. The remarkable reversibility of this thermally activated catalyst allows for multiple reprocessing cycles while effectively maintaining a creep-free state at service temperature.

Conventionally, thermosets exhibit robust mechanical properties and high stability due to their chemically cross-linked structure. However, they cannot be reshaped without undergoing degradation. In contrast, thermoplastics, which are linear or lightly branched polymers, can be reprocessed multiple times due to their reversible physical bonds, but the lack of covalent cross-linking limits their structural integrity for high-performance applications.^1^ Bridging both scenarios, covalent adaptable networks (CANs) combine the toughness of the first, with the malleability of the latter.^2^ CANs contain dynamic covalent bonds that exhibit on-demand chain rearrangement in response to external stimuli.^3,4^ The scope of molecular network rearrangements in CANs includes Diels–Alder reactions, associative addition/elimination exchange of imines or hydrazones, and disulfide exchange, among others.^5^

Despite their significant potential, one of the main challenges for the industrial implementation of CANs lies in the conflicting structural and chemical properties that are required to ensure sufficiently low viscosities during reprocessing while preventing minimal network relaxation during use.^6^ To accomplish this, materials with reversible bonds have to fulfill two prerequisites. First, the reversible bond exchange should be activated only above the service temperature and well before material degradation. Second, it is necessary to have a sufficiently low exchange rate at lower temperatures to ensure structural stability and prevent irreversible deformation (i.e., creep) during the service life.^7^ Hence, it is essential to attain an in-depth understanding of how chemical reactions can be effectively activated and controlled within polymer networks.

In a recent minireview, Winne and Du Prez provided a comprehensive overview of diverse chemical design strategies to combine creep resistance while keeping dynamism in CANs.^6^ These strategies include the structural control of the permanent and dynamic parts,^8^ the use of protecting groups,^9^ phase separation,^10,11^ promotion/inhibition of neighboring functional groups,^12−14^ and the addition of latent catalysts.^15,16^ In particular, the use of the latter has emerged as a prominent area of research interest since the dynamic exchange can be precisely controlled by using a small amount of additive within the polymer matrix. The catalyst is added as an ionic organic salt, which upon activation by either light or temperature will release the catalytic active species.

Specifically, quaternary ammonium salts exhibit distinct catalytic behavior by displaying basic properties upon liberation of a tertiary amine.^17,18^ Originally employed to achieve temporal and thermal control of the curing reaction processes of diverse formulations,^18−21^ and more recently to enhance the durability and stability of PU adhesives,^22^ this type of organocatalyst has garnered attention in polymer chemistry due to their facile and scalable synthesis, making their use suitable for industrial applications. More recently, this concept has been extended to trigger the dynamic exchange in CANs: Schlögl and co-workers showed the promotion of transesterification reactions by the thermal activation of a latent base, 1,5,7-triazabicyclo[4.4.0]dec-5-enylcyanoacetate (TBD-CA), in a cross-linked thiol–ene polymer network.^23^ The organic salt dissociation occurred at 130 °C, which also triggered the decarboxylation of the acid, leading to the irreversible release of the catalytic TBD. This group has also shown that a latent catalyst activation can be achieved by light. They reported the use of the quaternary ammonium salt 1,5,7-triazabicyclo[4.4.0]dec-5-enyltetraphenyl borate (PLB-TBD), which undergoes a radical cleavage of the anion upon UV exposure, releasing TBD, the active catalyst.^24^ In a related publication, Kim and co-workers showed that the transesterification of a similar cross-linked network can be triggered by using a triphenylsulfonium triflate photoacid generator (TPS-PAG) that is thermally stable.^16^ This catalyst, under UV exposure, undergoes an irreversible radical activation, releasing triflic acid and subsequently triggering the dynamic exchange. In all these cases, on-demand catalysis is achieved, offering creep resistance at service temperature. Nevertheless, the reported latent catalysts undergo irreversible reactions, which prevent multiple reprocessing or self-healing events.

Existing literature on tetraphenylborate salts has highlighted their efficacy as photobase generators (PBGs), being capable of generating basic species in situ upon UV irradiation (amines/amidines or guanidines).^25^ Specifically, Wang et al. proposed the photogeneration of TBD from its tetraphenylborate salt (H·TBD:BPh_4_) through a photoinduced proton transfer reaction. The photogenerated strong base was employed to catalyze the anionic ring-opening polymerization and cross-linking of ester-based polymeric materials. Nonetheless, the photoactivation of H·TBD:BPh_4_ is irreversible, resulting in the permanent release of the base, alongside photocleaved products. More recently, Serra and co-workers introduced thermal activation as an alternative method for activating a similar catalyst in a *trans*-thiocarbamoylation process in thiourethane-based networks.^19,21^ Nevertheless, the high temperature used leads to irreversible catalyst degradation.

Disulfide bonds are known for their ultrafast exchange, promoted by basic catalysts, which enables the material reprocessing.^26,27^ However, in the presence of free bases, a significant limitation arises from the potential network relaxation and irreversible deformation due to their rapid exchange, even at room temperature. To tackle this challenge, we devised a strategy to control the dynamic bond rate by incorporating a latent underlying catalyst into the network. In contrast to other dual organocatalysts based on ionic mixtures of Brønsted acids and bases,^17^ we envisaged that tetraphenylborate ammonium salts could act solely as base catalysts upon thermal-induced proton dissociation. We hypothesized that unlike the irreversible decomposition of the ensuing tetraphenylborate radical anion under photoexcitation, thermal treatment of ammonium:BPh_4_ should promote clean and reversible proton shuttling. Noticeably, the reversibility of this particular catalyst has remained unexplored, rendering it a highly compelling subject for our investigation.

In this work, we study the use of tetraphenylborate tetramethyl guanidinium salt (**TPB:TMG**) as a basic thermally reversible catalyst for catalyzing disulfide exchange in PU networks. By maintaining the catalyst in a latent state (low temperatures), we effectively arrest the disulfide exchange, thereby preventing creep deformation. Nevertheless, upon reaching the dissociation temperature and activating the switch, the network allows for reprocessability at elevated temperatures (Figure 1). A similar behavior was already described in polyester vitrimers by employing Lewis acids; indeed, Reynaud and co-workers reported epoxy vitrimers containing dynamic ester bonds and demonstrated that their reworkability at elevated temperatures could be achieved by introducing a Lewis catalyst without affecting the curing process occurring at room temperature.^28^

**Figure 1:**
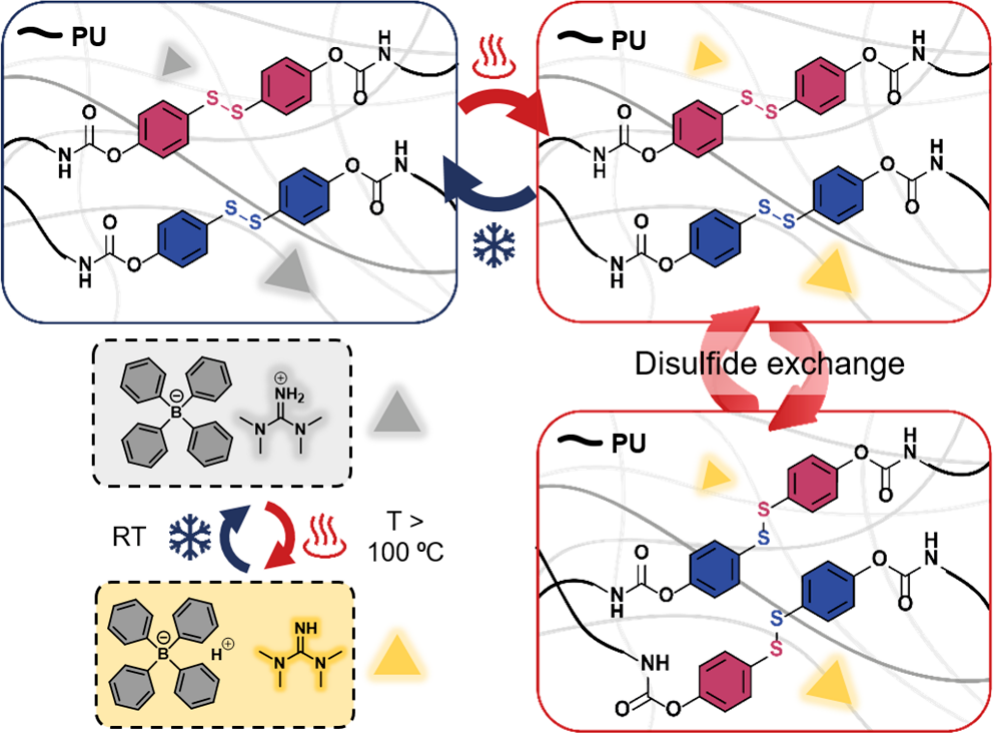
Representative disulfide exchange thermally activated by **TPB:TMG**, a thermally reversible catalyst.

As a proof of concept, we selected low *T*_g_ polyurethanes as a model network due to their industrial interest and the potential to tailor the properties.^29^ Disulfide bonds were incorporated into the network design because of their orthogonal chemistry and fast exchange mechanism, especially in the presence of free amines.^26,27,30,26^ We first synthesized a set of films containing bases with different p*K*_a_s by mixing trifunctional (branched) polypropylene glycol (PPG, *M*_n_ = 3740 g·mol^–1^) (**1**) and bifunctional hexamethylene isocyanate (HDI). Next, bis(4-hydroxyphenyl) disulfide and a base as additive were added to the formulation to obtain cross-linked polyurethane films, **PU-1**, incorporating the aromatic disulfide and a base catalyst (see Scheme S1). FTIR spectroscopy was employed to follow the prepolymer formation and the subsequent curing process. More information about the preparation of the PU networks as well as FTIR and DSC characterization of the materials is given in the Experimental Section in the SI (Figures S1–S3).

Two strong bases (DBN and TMG) and one milder base (DMAP) were chosen to study their catalytic performance in triggering the dynamic exchange. Stress relaxation measurements evidenced that the material containing TMG as a permanently active base relaxed more rapidly (Figure S4). Similarly, molecular model reactions revealed rapid disulfide exchange with no side reactions observed (Figure S5).

Once TMG was identified as the fastest basic catalyst, a tetraphenylborate tetramethyl guanidinium salt (**TPB:TMG**) was chosen as the thermally reversible catalyst. We prepared the ionic salt following a previously reported procedure for a related BPh_4_ ammonium salt.^25^ After successfully confirming the preparation of the catalyst, we investigated its thermal reversibility by ^1^H, ^11^B, and FTIR spectroscopy (Figures 2 and S6 and S7) and the stability by TGA (Figure S8). At 25 °C the organic salt dissolved in DMSO-*d*_6_ appears fully protonated, as evidenced by the multiplicity of the aromatic signals (δ 6.75–7.25 ppm) and, especially, the singlet at δ 7.75 ppm corresponding to the guanidinium NH_2_. The spectrum recorded at higher temperatures (from 25 to 120 °C) shows a gradual upfield shift of the labile proton, from 7.75 ppm (25 °C) to 7.50 ppm (120 °C), thus suggesting the shielding effect from the complexation, presumably with DMSO, a coordinating solvent.^31^ This is also evidenced by the splitting of the aromatic signals. More importantly, after cooling to 25 °C the spectra reverted to the original, thus indicating the stability of the catalyst and the reversibility of the process.

**Figure 2:**
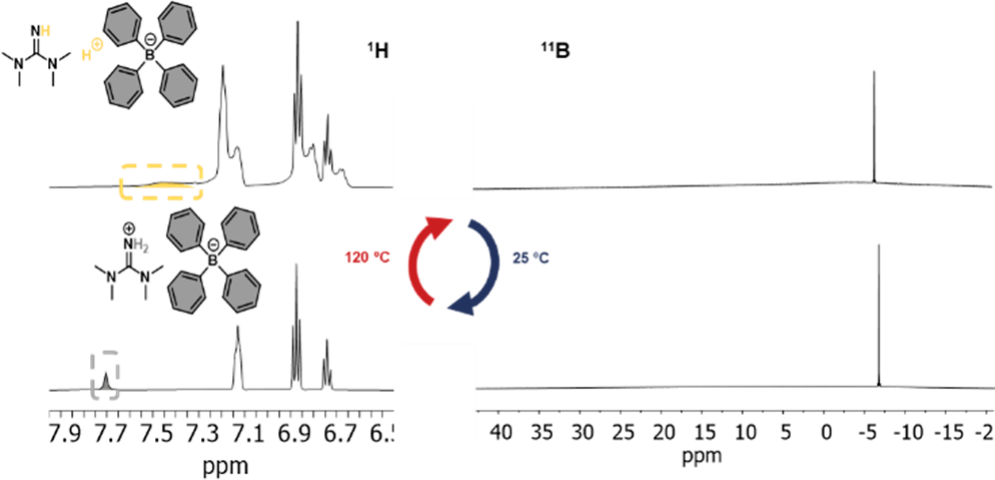
^1^H and ^11^B NMR spectra recorded at 25 and 120 °C of thermally reversible **TPB:TMG**. Reversibility is assessed upon several cycles of heating/cooling.

Additionally, FTIR spectroscopy conducted at 25 and 120 °C, and again at 25 °C, evidenced an increase in N–H stretch vibrations (between 3300 and 3500 cm^–1^) at 120 °C. This suggests the presence of additional N–H vibrations, presumably a consequence of partial proton complexation (Figure S7a). FTIR spectroscopy also revealed the degradation of the salt at 200 °C (Figure S7b).

Further evidence supporting the absence of the thermally reversible organocatalyst degradation at 120 °C is provided by ^11^B NMR spectroscopy: upon multiple cycles of heating/cooling, the peak corresponding to the borate at around −6.5 ppm is maintained, proving the stability of the BPh_4_^–^ counterion and no formation of borane species.

Previous literature has suggested that the latent catalyst activation was nonreversible, both when occurring photochemically and thermally.^19,21,25^ Nonetheless, our experiments conducted at 120 °C suggest the thermally reversible nature of the catalyst. It may be noted that these findings are not necessarily in conflict with the already cited literature, as the irreversible activation always occurred above 120 °C. To demonstrate the stability of the thermally reversible catalyst over time, a solution of the catalyst was monitored over 24 h at 120 °C, and aliquots were taken after 30 min and 24 h. Interestingly, at the examined temperature, ^1^H NMR did not exhibit any change (Figure S9). We then repeated the same experiments at 180 °C (the temperature at which thermal activation of the catalyst has previously been described in the literature). In that case, additional peaks appeared in the aromatic range after just 30 min (Figure S10), demonstrating that the stability of the catalyst is compromised at this temperature. Our experiments thus validated the reversibility of the thermally reversible catalyst over time at our operational temperature of 120 °C. To compare the effect of the permanently active base catalyst versus a latent thermally reversible one in the networks, we included free **TMG** and **TPB:TMG** in a 2% mol ratio in **PU-1**.

Next, we assessed the dynamic properties of aromatic disulfides in the presence of both catalysts (**TMG** and **TPB:TMG**), and stress relaxation measurements were conducted on the prepared **PU-1** films. The stress relaxation curves of the **PU-1** materials are illustrated in Figure 3a–c. The incorporation of 2 mol % **TMG** significantly improved the dynamicity of the network. The relaxation times observed were consistently shorter across the entire temperature range examined (80–120 °C). Next, we tested the dynamicity of the system in the presence of the thermally reversible catalyst (**TPB:TMG**). Curiously, at temperatures below 100 °C the stress relaxation times were considerably longer than in **PU-1-TMG** materials; Conversely, stress relaxation times at temperatures exceeding 100 °C were comparable with those observed in the **TMG**-containing materials. Indeed, a sharp drop in relaxation time can be observed when comparing the **PU-1-TPB:TMG** network at 80 and 100 °C (from an average of ≈6360 s at 80 °C to ≈180 s at 100 °C). These results suggest an acceleration of the dynamic behavior, which agrees with the results observed by NMR for the thermal activation of **TPB:TMG**.

**Figure 3:**
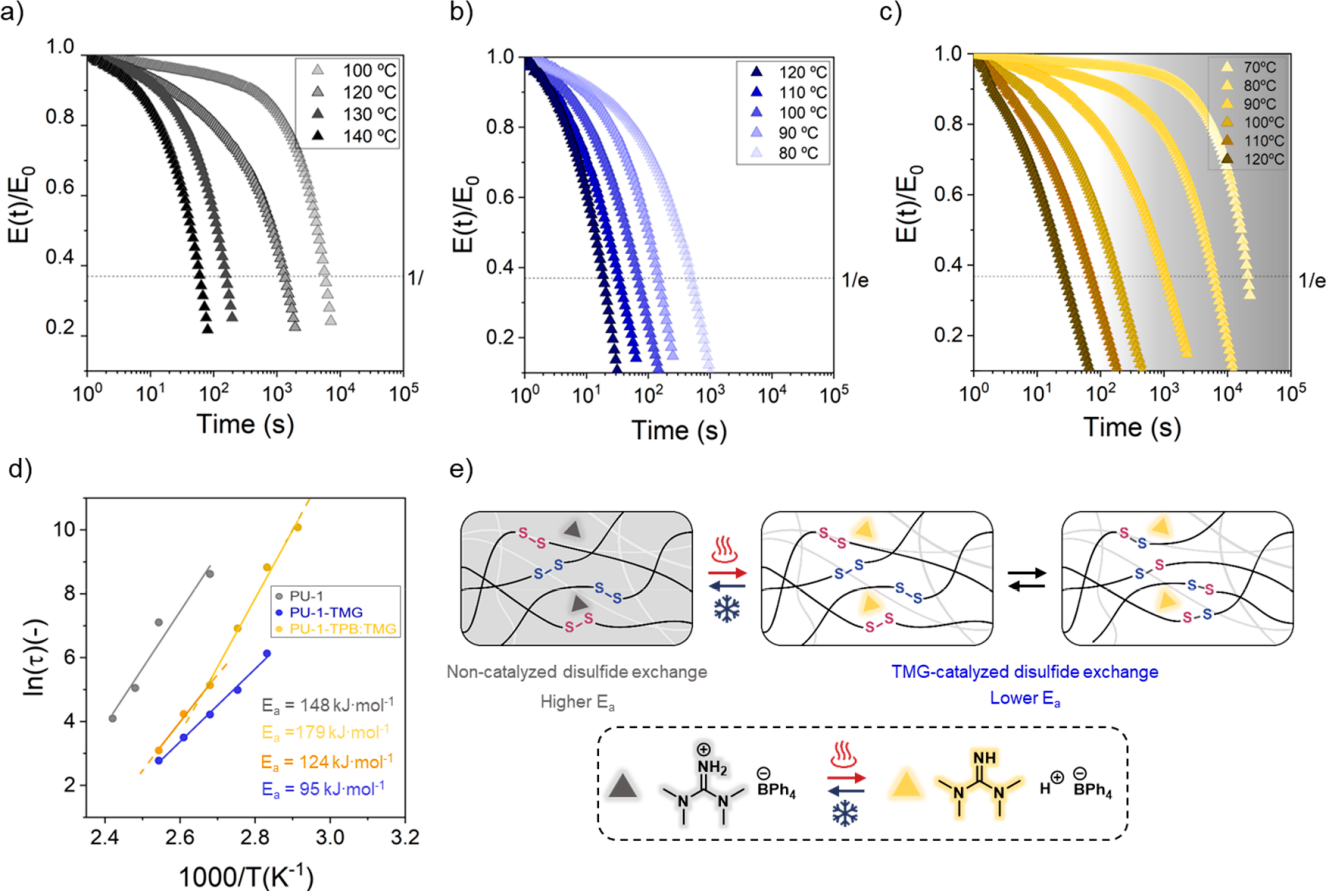
Stress relaxation and Arrhenius plot curves for the **PU-1** series: (a) **PU-1**, (b) **PU-1-TMG**, and (c) **PU-1-TPB:TMG**. (d) Arrhenius plot of **PU-1**: gray; **PU-1-TMG**, blue; **PU-1-TPB:TMG**, yellow. The latter illustrates with a dashed line the two slopes observed in the latent and active states. (e) Schematic representation of the base-catalyzed disulfide exchange upon thermally reversible activation.

Regarding the activation energies (*E*_a_), a temperature dependency was observed, varying depending on the presence or absence of the catalyst (Arrhenius plot, Figure 3d). The *E*_a_ and the relative errors obtained from the Arrhenius plots are summarized in Table S1. The activation energy for the pristine material was calculated as 148 kJ·mol^–1^ (PU-1), while the *E*_a_ was found to be much lower for the TMG-containing PUs (95 kJ·mol^–1^). More interestingly, the *E*_a_ of **PU-TPB:TMG**-containing material did not show the same linear trend along the temperature regime explored. **PU-1-TPB:TMG** exhibited two different linear slopes (80 °C ≤ *T* ≤ 100 and 100 °C ≤ *T* ≤ 120 °C), and two different *E*_a_ could be calculated. In the first range (when the activation of the catalyst is negligible), the average activation energy was 179 kJ·mol^–1^, while at higher temperatures, the *E*_a_ calculated was 124 kJ·mol^–1^. Interestingly, above 100 °C, when the thermally reversible organocatalyst dissociation becomes noticeable, the stress relaxation times of **PU-1-TPB:TMG** align with those of the **PU-1-TMG** and the *E*_a_ approaches the values obtained for **PU-1-TMG**.

Overall, **PU-1-TPB:TMG** exhibited two distinct viscoelastic regimes based on the availability of the thermally reversible catalyst. The change in the trend can be attributed to the progressive activation of the switch with increasing temperature, leading to an enhanced disulfide exchange rate within the network. As a result of the activation of the thermally reversible organocatalyst, the stress relaxation times as well as the *E*_a_ values approach those found in the materials containing the permanent active base.^6,7,9^

Next, the deformation behavior of **PU-1-TPB:TMG** at various temperatures was examined, in comparison to both the **PU-1** reference and the **PU-TMG** material. Creep experiments were performed by applying a constant shear stress of 5 kPa for 3000 s at 60, 100, and 120 °C, and the resulting strain was monitored as a function of time (Figure 4). Initially, at 60 °C no irreversible deformation was observed for the **PU-TPB:TMG** material as well as for the reference sample (**PU-1**). Conversely, the **PU-1-TMG** material exhibited a low creep. As the temperature increased to 100 °C, a more pronounced difference in the irreversible deformation between the **PU-1-TPB:TMG** material and its reference (**PU-1-TMG**) was observed. Specifically, the **PU-1-TPB:TMG** samples displayed reduced creep, while the **PU-1-TMG** samples exhibited significant irreversible deformation (Figure 4b–d). Moreover, it should be noted that the reference materials did not show any irreversible deformation at any of the investigated temperatures. This trend was further confirmed by conducting additional creep experiments at 120 °C.

**Figure 4:**
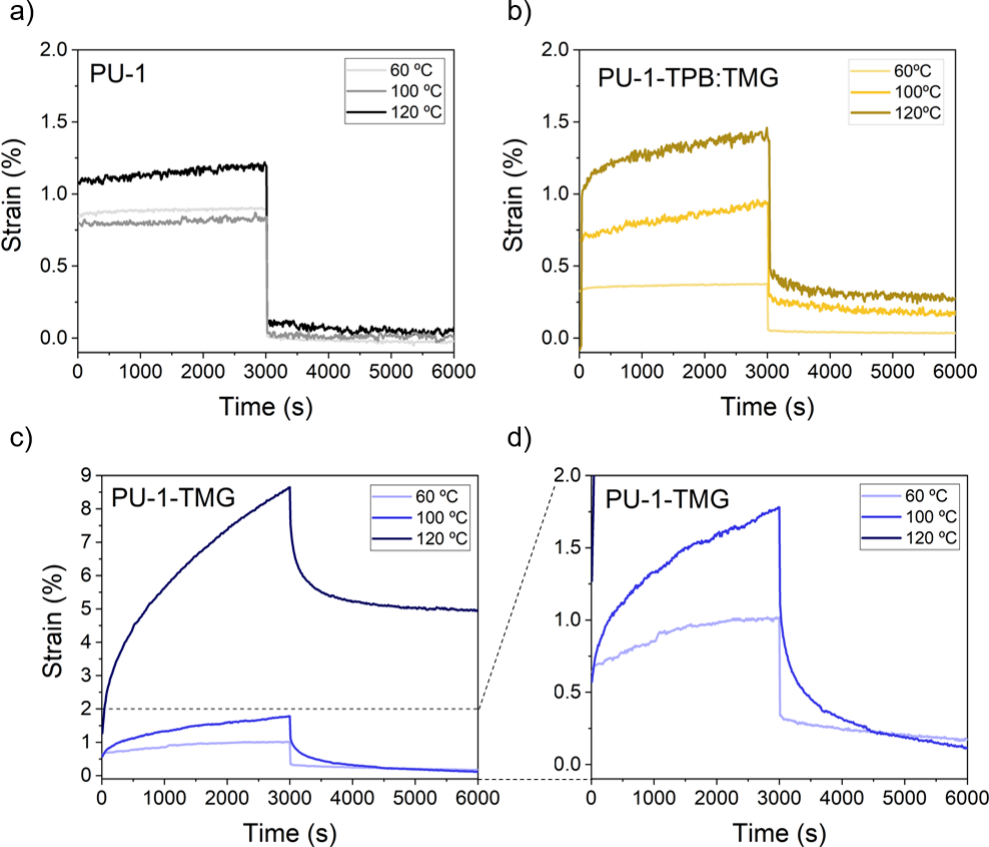
Creep experiments performed on the **PU-1** materials series. (a) Creep measurements of **PU-1** (pristine) at 60, 100, and 120 °C. (b) Creep measurements of **PU-1-TPB:TMG** at 60, 100, and 120 °C. (c) Creep measurements of **PU-1-TMG** at 60, 100, and 120 °C. (d) Zoom of creep measurements of **PU-1-TMG** at 60, 100, and 120 °C (same *y* scale as in (a) and (b)).

Similar conclusions were also drawn from the analysis of compliance changes with temperature within the strain recovery curve (Figures S11 and S12). The nonrecoverable compliance (*J*_nr_), or final strain, is a parameter that refers to the component of the total compliance that is not recoverable after the removal of an applied load. Thus, it measures the residual deformation of a material and can be used to quantify the creep. A comparison of the *J*_nr_ values within the **PU-1** series, between 60 and 120 °C, reveals that the increment in *J*_nr_ for **PU-1-TMG** is considerably higher than those of **PU-1** and **PU-1-TPB:TMG**. Precisely, the *J*_nr_ value of the **PU-1-TMG** increased by over 2 orders of magnitude between 60 and 120 °C, while the *J*_nr_ increase of **PU-1-TPB:TMG** was just a bit over 1 order of magnitude (Figure S11), in the same range of temperature. The values of *J*_nr_ at different temperatures are resumed in the Supporting Information (Table S2).

Above, we have demonstrated the reversibility of the thermally reversible organocatalyst by ^1^H NMR spectroscopy; however, to further corroborate the transient nature of its activity within the material, we conducted creep experiments at different temperatures, ranging from 60 °C (no creep) to 120 °C (evidence of creep) and subsequently returning to 60 °C. This investigation aimed to evaluate the efficacy of the thermally reversible catalyst, which remains only transiently active in the material. Creep experiments were conducted in a sequential manner at two distinct temperatures, 60 and 120 °C, and then reverted back to 60 °C; each temperature cycle was performed 4 times for 3000 s, and the obtained results are illustrated in Figure 5. The strain curves at 60 °C before and after the treatment at 120 °C displayed identical behavior along the 4 cycles. Furthermore, the strain curves of the four cycles at 120 °C exhibited an identical trend. Only in the final measurement was any reduction of viscoelastic deformation (i.e., creep) observed, although even in this case the change was negligible. This observation led us to conclude that activation of **TPB:TMG** within the material can be considered reversible.

**Figure 5:**
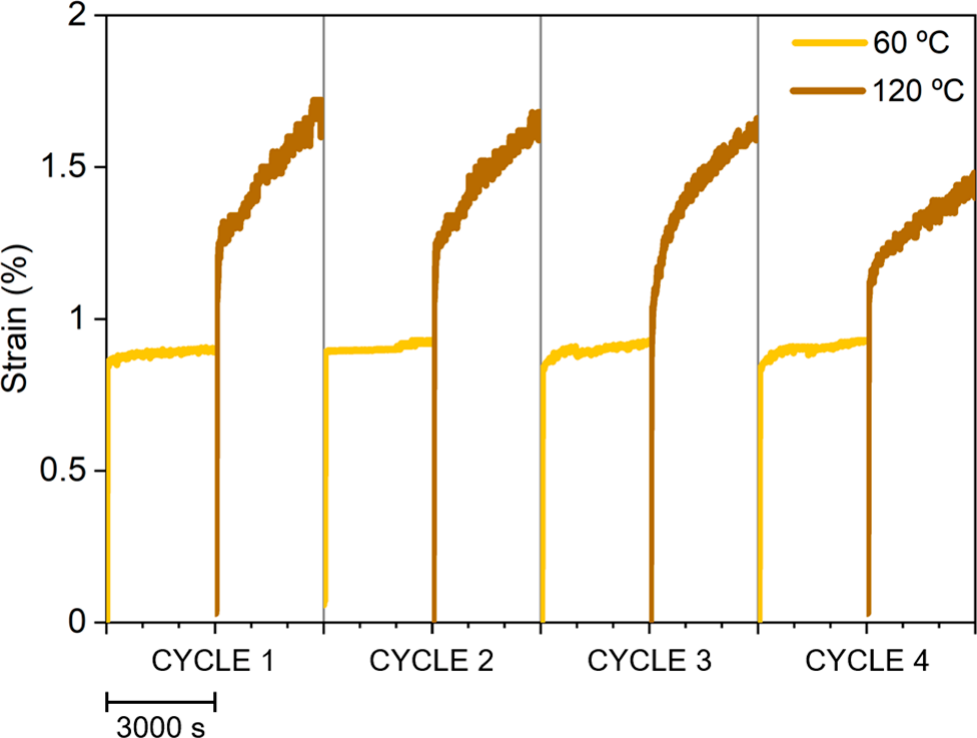
Creep experiments performed on **PU-1-TPB:TMG**. Each measurement has a duration of 3000 s.

The final aim of this study was to assess the effectiveness of the reversible organocatalyst in preventing creep in elastomeric vitrimers while maintaining their reprocessability. Hence, reprocessing experiments were conducted on both PU series (**PU**, **PU-TMG** and **PU-TPB:TMG**), and the results were compared. The samples were placed in a circular mold of 3 cm in diameter and 2 mm thick and compressed in a hot press at 100 °C ≤ T ≤ 120 °C and pressure of 3 MPa, for times ranging from 15 to 30 min. The outcomes for both PU-1 series are depicted in Figure 6a. In both cases, **PU-TMG** and **PU-TPB:TMG** could be successfully reprocessed, resulting in homogeneous films. Conversely, attempts to reprocess the reference materials under the conditions indicated did not yield any films. Dynamic mechanical analysis (DMA) was conducted on the reference **PU-1** and on the corresponding samples after the first reprocessing step (**PU-1-TMG** and **PU-1-TPB:TMG**). The results are presented in Figure 6b. For **PU-1-TMG** there was a significant difference in the storage modulus after the first reprocessing step across the examined temperature range. In contrast, for **PU-1-TPB:TMG**, the storage modulus was fully preserved at the temperature studied (up to 100 °C), demonstrating that full reprocessability was achieved by replacing the permanent active TMG with the thermally reversible catalyst. Furthermore, FTIR analysis did not reveal any discernible change in the **PU-1-TMG** and **PU-1-TPB:TMG** samples before and after the reprocessing (Figure S13).

**Figure 6:**
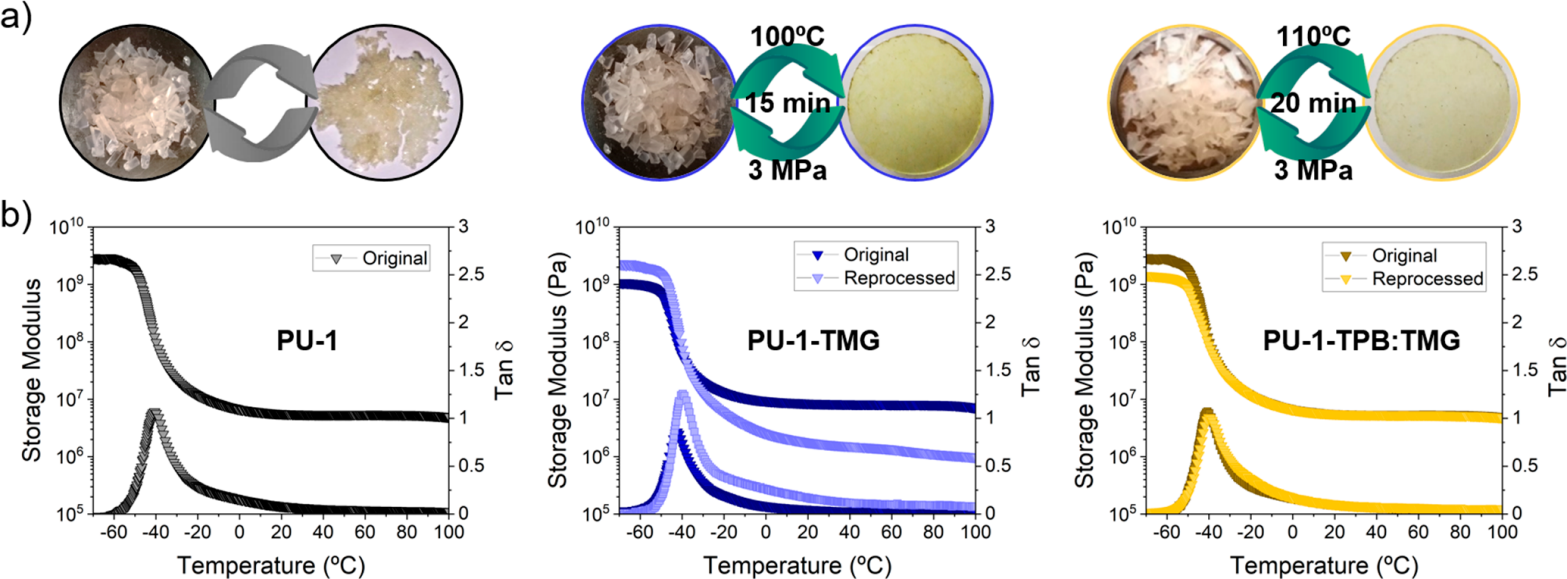
Reprocessing of the **PU-1** series. (a) Conditions at which the reprocessing was performed (for **PU-1** series). (b) Dynamic mechanical analysis (DMA) of **PU-1** (pristine), **PU-1-TMG** and a comparison with the reprocessed, and **PU-1-TPB:TMG** and a comparison with the reprocessed.

To further corroborate the effectiveness of the thermally reversible organocatalyst in selectively activating the disulfide dynamic bonds, a different formulation was prepared. In this formulation, a shorter triol was employed to evaluate whether the thermally reversible catalyst was effective in a material with higher cross-linking density. Thus, ALCUPOL triol (*M*_n_ = 1050 g·mol^–1^) (**2**) and bifunctional hexamethylene isocyanate (HDI) were mixed, and the prepolymer **2** was obtained (S1). Next, the bis(4-hydroxyphenyl) disulfide and the base catalysts were added to obtain the cross-linked polyurethane film series, **PU-2**. The scheme of the synthesis and the FTIR and DSC characterization are reported in the Experimental Section (S1–S3).

Differential scanning calorimetry (DSC) analysis revealed a slightly higher glass transition temperature (*T*_g_) compared to that in the previous PU series (Figure S3). The results obtained for the **PU-2** series closely mirrored those of the **PU-1** series, with similar stress relaxation times in the range of temperature measured in all the **PU-2** formulations (Figure S14a–c). The Arrhenius plot for **PU-2-TPB:TMG** revealed a similar nonlinear trend in activation energy observed in **PU-1-TPB:TMG**, albeit shifted toward higher temperatures (Figure S14d). In this case, the *E*_a_ was calculated to be 148 kJ·mol^–1^ between 80 and 120 °C and it decreased to 87 kJ·mol^–1^ between 120 and 140 °C. We hypothesized that a more efficient activation of the thermally reversible organocatalyst at elevated temperatures was essential to approach the stress relaxation times observed in **PU-2-TMG**, particularly in the context of a more highly cross-linked material. The *E*_a_ values and their relative errors obtained from the Arrhenius plots are summarized in Table S1.

Creep experiments conducted on the **PU-2** series confirmed the findings obtained of the **PU-1** series. Specifically, the reference material (**PU-2**) exhibited a solidlike behavior within the studied temperature range (60–120 °C). Additionally, **PU-2-TMG** showed significantly higher irreversible deformation when compared to **PU-TPB: TMG**, as illustrated in Figures S12 and S15. Also, an impressive improvement in the reprocessability of the thermally reversible catalyst-containing material (**PU-2-TPB:TMG**) was observed compared to **PU-2-TMG**, consistent with the findings from the **PU-1** series (Figure S16). However, full reprocessability was not achieved, and it was surmised that the limited chain mobility in this specific formulation at the studied temperatures contributed to incomplete reprocessability. Overall, the experiments conducted on the **PU-2** networks provided further confirmation of the thermally reversible catalyst’s effectiveness in fine-tuning the rheological properties of the vitrimer material.

In conclusion, in this work, we demonstrated the effectiveness of a thermally reversible organocatalyst as a versatile approach for modifying specific properties in vitrimers. More precisely, our findings showcase the possibility of preventing creep at service (lower) temperatures while maintaining the reprocessability in PU-disulfide vitrimers at elevated temperatures. Cyclic creep measurements performed at service and reprocessing temperatures confirmed the reversibility of the tetraphenyl borate salt as a thermally reversible catalyst. Interestingly, the inactivity or activity of the reversible catalyst unveiled two distinct linear trends in the activation energy: at lower temperatures, both the series of materials studied exhibit higher values of *E*_a_, while at higher temperatures, the activation energy approached values similar to those found in the material containing the permanently active catalyst.^7^

This ability to selectively activate the disulfide dynamic bonds through the reversible thermally activated catalyst offers a valuable avenue for precisely tailoring the material’s behavior and opens up possibilities for diverse applications in responsive and adaptable polymer systems.

## Supporting Information Available

Additional experimental details, materials, and methods, including photographs of the experimental setup (PDF)

## References

[ref1] Jehanno C.; Sardon H. Dynamic polymer network points the way to truly recyclable plastics. Nature 2019, 568, 467–468. 10.1038/d41586-019-01209-3. 31011182

[ref2] Zheng N.; Xu Y.; Zhao Q.; Xie T. Dynamic Covalent Polymer Networks: A Molecular Platform for Designing Functions beyond Chemical Recycling and Self-Healing. Chem. Rev. 2021, 121 (3), 1716–1745. 10.1021/acs.chemrev.0c00938. 33393759

[ref3] McBride M. K.; Worrell B. T.; Brown T.; Cox L. M.; Sowan N.; Wang C.; Podgorski M.; Martinez A. M.; Bowman C. N. Enabling Applications of Covalent Adaptable Networks. Annu. Rev. Chem. Biomol. Eng. 2019, 10 (1), 175–198. 10.1146/annurev-chembioeng-060718-030217. 30883213

[ref4] Zhang V.; Kang B.; Accardo J. V.; Kalow J. A. Structure-Reactivity-Property Relationships in Covalent Adaptable Networks. J. Am. Chem. Soc. 2022, 144 (49), 22358–22377. 10.1021/jacs.2c08104. 36445040 PMC9812368

[ref5] Winne J. M.; Leibler L.; Du Prez F. E. Dynamic Covalent Chemistry in Polymer Networks: A Mechanistic Perspective. Polym. Chem. 2019, 10 (45), 6091–6108. 10.1039/C9PY01260E.

[ref6] Van Lijsebetten F.; Debsharma T.; Winne J. M.; Du Prez F. E. A Highly Dynamic Covalent Polymer Network without Creep: Mission Impossible?. Angew. Chem. Int. Ed 2022, 61 (48), na 10.1002/anie.202210405. 36161440

[ref7] Obadia M. M.; Jourdain A.; Cassagnau P.; Montarnal D.; Drockenmuller E. Tuning the Viscosity Profile of Ionic Vitrimers Incorporating 1,2,3-Triazolium Cross-Links. Adv. Funct. Mater. 2017, 27 (45), 1703258 10.1002/adfm.201703258.

[ref8] Li L.; Chen X.; Jin K.; Torkelson J. M. Vitrimers Designed Both To Strongly Suppress Creep and To Recover Original Cross-Link Density after Reprocessing: Quantitative Theory and Experiments. Macromolecules 2018, 51 (15), 5537–5546. 10.1021/acs.macromol.8b00922.

[ref9] Van Lijsebetten F.; De Bruycker K.; Spiesschaert Y.; Winne J. M.; Du Prez F. E. Suppressing Creep and Promoting Fast Reprocessing of Vitrimers with Reversibly Trapped Amines. Angew. Chem. Int. Ed 2022, 61 (9), na 10.1002/anie.202113872. 34981887

[ref10] Lessard J. J.; Scheutz G. M.; Sung S. H.; Lantz K. A.; Epps T. H.; Sumerlin B. S. Block Copolymer Vitrimers. J. Am. Chem. Soc. 2020, 142 (1), 283–289. 10.1021/jacs.9b10360. 31794219

[ref11] Ricarte R. G.; Shanbhag S. Unentangled Vitrimer Melts: Interplay between Chain Relaxation and Cross-Link Exchange Controls Linear Rheology. Macromolecules 2021, 54 (7), 3304–3320. 10.1021/acs.macromol.0c02530.

[ref12] Fortman D. J.; Brutman J. P.; Cramer C. J.; Hillmyer M. A.; Dichtel W. R. Mechanically Activated, Catalyst-Free Polyhydroxyurethane Vitrimers. J. Am. Chem. Soc. 2015, 137 (44), 14019–14022. 10.1021/jacs.5b08084. 26495769

[ref13] Fortman D. J.; Brutman J. P.; Hillmyer M. A.; Dichtel W. R. Structural Effects on the Reprocessability and Stress Relaxation of Crosslinked Polyhydroxyurethanes. J. Appl. Polym. Sci. 2017, 134 (45), 44984 10.1002/app.44984.

[ref14] Van Lijsebetten F.; Spiesschaert Y.; Winne J. M.; Du Prez F. E. Reprocessing of Covalent Adaptable Polyamide Networks through Internal Catalysis and Ring-Size Effects. J. Am. Chem. Soc. 2021, 143 (38), 15834–15844. 10.1021/jacs.1c07360. 34525304

[ref15] Worrell B. T.; McBride M. K.; Lyon G. B.; Cox L. M.; Wang C.; Mavila S.; Lim C.-H.; Coley H. M.; Musgrave C. B.; Ding Y.; Bowman C. N. Bistable and Photoswitchable States of Matter. Nat. Commun. 2018, 9 (1), 2804 10.1038/s41467-018-05300-7. 30022053 PMC6052001

[ref16] Park J.; Song H. Y.; Choi S.; Ahn S.; Hyun K.; Kim C. B. Spatiotemporal Vitrimerization of a Thermosetting Polymer Using a Photo-Latent Catalyst for Transesterification. J. Mater. Chem. A 2022, 10 (12), 6475–6480. 10.1039/D1TA10841G.

[ref17] Basterretxea A.; Jehanno C.; Mecerreyes D.; Sardon H. Dual Organocatalysts Based on Ionic Mixtures of Acids and Bases: A Step Toward High Temperature Polymerizations. ACS Macro Lett. 2019, 8 (8), 1055–1062. 10.1021/acsmacrolett.9b00481. 35619485

[ref18] Kobayashi A.; Kobayashi F.; Ebina T.; Ishii R.; Nakamura T.; Nge T. T.; Yamada T.; Shiraishi A.; Yamashita T. Effect of Thermal Base Generators on the FRP Fabrication with Glycol-Lignin. J. Photopol. Sci. Technol. 2018, 31 (1), 101–106. 10.2494/photopolymer.31.101.

[ref19] Gamardella F.; Ramis X.; De la Flor S.; Serra À. Preparation of Poly(Thiourethane) Thermosets by Controlled Thiol-Isocyanate Click Reaction Using a Latent Organocatalyst. React. Funct. Polym. 2019, 134, 174–182. 10.1016/j.reactfunctpolym.2018.11.019.

[ref20] Konuray A. O.; Fernández-Francos X.; Ramis X. Latent Curing of Epoxy-Thiol Thermosets. Polymer 2017, 116, 191–203. 10.1016/j.polymer.2017.03.064.

[ref21] Gamardella F.; Muñoz S.; De la Flor S.; Ramis X.; Serra A. Recyclable Organocatalyzed Poly(Thiourethane) Covalent Adaptable Networks. Polymers 2020, 12 (12), 2913 10.3390/polym12122913. 33291704 PMC7761908

[ref22] Vallejos S.; Trigo-López M.; Sardon H.; González-Martín J. M.; González-Moreno S.; García J. M. Metal-Free Organocatalysts for High Hydrolytic Stability Single Component Polyurethane Adhesives and Their Application in Decorative Insulation Facades Manufacturing. Construction and Building Materials 2023, 400, 132643 10.1016/j.conbuildmat.2023.132643.

[ref23] Reisinger D.; Kriehuber M. U.; Bender M.; Bautista Anguís D.; Rieger B.; Schlögl S. Thermally Latent Bases in Dynamic Covalent Polymer Networks and Their Emerging Applications. Adv. Mater. 2023, 35, 2300830 10.1002/adma.202300830. 36916976

[ref24] Reisinger D.; Kaiser S.; Rossegger E.; Alabiso W.; Rieger B.; Schlögl S. Introduction of Photolatent Bases for Locally Controlling Dynamic Exchange Reactions in Thermo Activated Vitrimers. Angew. Chem., Int. Ed. 2021, 60 (26), 14302–14306. 10.1002/anie.202102946. 33929092

[ref25] Sun X.; Gao J. P.; Wang Z. Y. Bicyclic Guanidinium Tetraphenylborate: A Photobase Generator and A Photocatalyst for Living Anionic Ring-Opening Polymerization and Cross-Linking of Polymeric Materials Containing Ester and Hydroxy Groups. J. Am. Chem. Soc. 2008, 130 (26), 8130–8131. 10.1021/ja802816g. 18528981

[ref26] Rekondo A.; Martin R.; Ruiz de Luzuriaga A.; Cabañero G.; Grande H. J.; Odriozola I. Catalyst-Free Room-Temperature Self-Healing Elastomers Based on Aromatic Disulfide Metathesis. Mater. Horiz. 2014, 1 (2), 237–240. 10.1039/C3MH00061C.

[ref27] Martin R.; Rekondo A.; Ruiz de Luzuriaga A.; Cabañero G.; Grande H. J.; Odriozola I. The Processability of a Poly(Urea-Urethane) Elastomer Reversibly Crosslinked with Aromatic Disulfide Bridges. J. Mater. Chem. A 2014, 2 (16), 5710 10.1039/c3ta14927g.

[ref28] Liu W.; Schmidt D. F.; Reynaud E. Catalyst Selection, Creep, and Stress Relaxation in High-Performance Epoxy Vitrimers. Ind. Eng. Chem. Res. 2017, 56 (10), 2667–2672. 10.1021/acs.iecr.6b03829.

[ref29] Bakkali-Hassani C.; Berne D.; Ladmiral V.; Caillol S. Transcarbamoylation in Polyurethanes: Underestimated Exchange Reactions?. Macromolecules 2022, 55 (18), 7974–7991. 10.1021/acs.macromol.2c01184.

[ref30] Nevejans S.; Ballard N.; Fernández M.; Reck B.; Asua J. M. Flexible Aromatic Disulfide Monomers for High-Performance Self-Healable Linear and Cross-Linked Poly(Urethane-Urea) Coatings. Polymer 2019, 166, 229–238. 10.1016/j.polymer.2019.02.001.

[ref31] Molchanov S.; Gryff-Keller A. Solvation of Amides in DMSO and CDCl _3_ : An Attempt at Quantitative DFT-Based Interpretation of ^1^ H and ^13^ C NMR Chemical Shifts. J. Phys. Chem. A 2017, 121 (50), 9645–9653. 10.1021/acs.jpca.7b10110. 29179531

