# Supplementary material for: Thermally Reversible Organocatalyst for the Accelerated Reprocessing of Dynamic Networks with Creep Resistance
Source: ACS Macro Lett. 2023 Nov 1;12(11):1536–42. doi: 10.1021/acsmacrolett.3c00544 (PMC10666533; doi:10.1021/acsmacrolett.3c00544)
Supplement: Supplementary file 1 — mz3c00544_si_002.pdf [file mz3c00544_si_002.pdf]

Supporting Information

Thermally-reversible organocatalyst for the accelerated reprocessing of dynamic networks with creep resistance

Giulia Vozzolo,[1] Marta Ximenis,[1,*] Daniele Mantione,[1,2] Mercedes Fernández[1] and Haritz Sardon[1,*]

[1] POLYMAT, University of the Basque Country UPV/EHU, Joxe Mari Korta Center, Avda. Tolosa 72, 20018 Donostia-San Sebastian, Spain.

[2] Ikerbasque, Basque Foundation for Science. 48013 Bilbao, Spain

Corresponding Author

* Haritz Sardon - POLYMAT, University of the Basque Country UPV/EHU, Joxe Mari Korta Center, Avda. Tolosa 72, 20018 Donostia-San Sebastian, Spain;
Orcid https://orcid.org/0000-0002-6268-0916;
Email: haritz.sardon@ehu.es
* Marta Ximenis - POLYMAT, University of the Basque Country UPV/EHU, Joxe Mari Korta Center, Avda. Tolosa 72, 20018 Donostia-San Sebastian, Spain;
Orcid https://orcid.org/0000-0002-6550-6307;
Email: marta.ximenis@polymat.eu

Experimental section

Materials

Hexamethylene diisocyanate (HDI, ≥98.0%), phenyl isocyanate (>98%), tetramethyl guanidine (TMG), 1,5-Diazabicyclo[4.3.0]non-5-ene (DBN), Diaminomethylpyridine (DMAP), anhydrous tetrahydrofuran (THF), anhydrous acetonitrile (ACN) were purchased from Aldrich and used as received. Bis(4-hydroxyphenyl) Disulfide (>98%), and Di-p-tolyl Disulfide (>98%) were received from TCI and used as received. Sodium tetraphenylborate was purchased from abcr and used as received. Poly(propylene glycol) (PPG) (MW = 3740g/ mol) was purchased from Bayer Materials Science and dried in the oven for 48 h at 60 ^{o}C prior to use. ALCUPOL (MW=1050 g/mol) was purchased by CIDETEC and dried in the oven for 48 h at 60 ^{o}C prior to use.

Instrumentation

Nuclear magnetic resonance (NMR) analyses were conducted on a Bruker Avance 300 (300 MHz) to measure proton spectra at 25 ^{o}C. The NMR spectra were measured in DMSO-d_6, and chemical shifts (δ) are presented in parts per million (ppm), relative to residual solvent as the internal standard. High-temperature experiments were conducted on a Bruker Avance 500 (500 MHz) to measure proton spectra at 25 ^{o}C <T<120 ^{o}C.

Fourier transform infrared spectroscopy. FT-IR spectra were obtained by FT-IR spectrophotometer (Nicolet 6700 FT-IR, Thermo Scientific Inc., USA) using attenuated total reflectance (ATR) technique (Golden Gate, Specac). Spectra were recorded between 4000–600 cm^{-1} with a spectral resolution of 4 cm^{-1}. All spectra were averaged over 32 scans.

Thermogravimetric analyses (TGA) were performed with a TGA/Q500 TA instrument under Nitrogen atmosphere in a platinum crucible at a heating rate of 10 ^{o}C.min^{-1} from 25 ^{o}C to 800 ^{o}C

Differential scanning calorimetry (DSC) analyses were performed with DSC 25 TA instruments under Nitrogen atmosphere at a heating and cooling rate of 10 ^{o}C.min^{-1}. Measurements were performed, depending on the sample, in a range of temperature from -70 ^{o}C to 100 ^{o}C.

Stress relaxation measurements. The experiments were performed to obtain the relaxation modulus G(t) using an ARES rheometer (Rheometrics) under the conditions indicated in each experiment, using a film tension fixture and 1% strain. Employed samples had a width between 3.0 and 4.0 mm, and a thickness between 0.5 and 1 mm.

The temperature dependence of relaxation time is described by the Arrhenius equation:

$$\tau(T) = \tau_0 \exp\left(\frac{E_a}{RT}\right) \quad (1)$$

where the activation energy was calculated.

Dynamic mechanical thermal analysis (DMTA) was conducted in tension mode in a Dynamic Mechanical Analyzer, Triton 2000 DMA (Triton Technology). Experiments were performed at a frequency of 1 Hz upon heating at 4 ^{o}C/min (from 25 to 120 ^{o}C) was used to determine the glass transition temperature T_g, the temperature-dependent tensile storage modulus, E' and the tensile loss modulus, E'', using rectangular shape samples. All the measurements were carried out with 0.010 of displacement. Samples employed for these measurements had a thickness between 0.50 and 1.0 mm and a width between 6 and 7 mm. All the samples were measured with a preset length of 5.00 mm.

Creep experiments at different temperatures (60-120 ^{o}C) were performed on an Anton Paar MCR 101 in parallel plate geometry using 8 mm sample disks. A constant force of 3 N was chosen. Additionally, in the first 180 s, no shear stress was applied. Afterward, a 5000 Pa shear stress was applied for 3000 s, followed by a recovery of 3000 s. Creep measurements were preceded by a time sweep measurement at 90 ^{o}C and a fixed frequency of 1 Hz for 30 minutes to remove possible thermal history.

Reprocessing experiments. The polymer was cut and broken into pieces of about 1 cm and placed into a circular mold for compression molding. This assembly was placed in a preheated compression press (90 to 120 ^{o}C) for 5 min with no pressure. Then the pressure was increased to 3 MPa and kept constant for the selected time (from 10 to 60 minutes). After 10 to 60 minutes of pressing in total, the sample was removed from the mold. The temperature and pressing time were adjusted according to the catalyst present in the material.

Synthetic procedures.

Synthesis of 1,1'-(disulfanediylbis(4,1-phenylene))bis(3-phenylurea)

4-Aminophenyl disulfide (5 mmol 1.24 g) was dissolved in the minimum amount of anhydrous DCM. Phenyl isocyanate (10 mmol 1.086 mL) was dissolved in the same amount of anhydrous DCM and then added to the solution dropwise. The mixture was stirred overnight at reflux. The precipitate was filtered off and washed with DCM. The white solid was collected and dried under vacuum. Yield: quantitative

Synthesis of the thermally-reversible organocatalyst (TPB:TMG)

The tetraphenylborate tetramethyl guanidinium salt (TPB:TMG) was synthesized according to a reported procedure.[1] To 2.5 mL of water slightly acidified with 1 mL of conc., 36% HCl solution, and 1.25 mL (10 mmol) of tetramethylguanidine (TMG) were added. Then, $NaBPh_4$ (11 mmol, 3.764 g) was solubilized in water and stirred until complete dissolution. The two aqueous solutions were mixed, forming a white salt as a precipitate. The salt was filtered, washed thoroughly with distilled water and MeOH, then recrystallized from a 4:1 mixture of MeOH and $CHCl_3$, filtered, and dried under mild temperature and vacuum.

*Synthesis of the tris-isocyanate-terminated pre-polymer (**1**)*

Dried PPG (3740 g mol^{-1}) (60 g, 16 mmol) and HDI (8.07 g, 48 mmol) were fed into a round bottom flask equipped with a stirrer and an N_2 inlet. The mixture was stirred for 8 hours at 80 ^{o}C and the reaction was monitored by FTIR spectroscopy. The resulting tris-isocyanate-terminated prepolymer was obtained as a colorless liquid and stored under N_2 at -18 ^{o}C.

*Synthesis of the tris-isocyanate-terminated pre-polymer (**2**)*

Dried ALCUPOL (R1610) (1050 g mol^{-1}) (16.8 g, 16 mmol) and HDI (8.07 g, 48 mmol) were fed into a round bottom flask equipped with a stirrer and a N_2 inlet. The mixture was stirred for 7 hours at 80 ^{o}C and the reaction was monitored by FTIR spectroscopy. The resulting tris-isocyanate-terminated prepolymer was obtained as a colorless liquid and stored under N_2 at -18 ^{o}C.

*Synthesis of the cross-linked polyurethane film (**PU-1**)*

Pristine **PU-1** film: (4-hydroxyphenyl) Disulfide (187.75 mg, 0.75 mmol) previously dissolved in 0.2 mL of anhydrous THF was mixed with the tris-isocyanate terminated pre-polymer (2.12 g, 0.5 mmol) and stirred vigorously. The mixture (**PU-1**) was degassed under vacuum and placed in an open mold. The material was cured under vacuum at room temperature and then at 40-50 ^{o}C overnight.

PU-1-TMG film: TMG was dissolved in anhydrous THF (11.5 mg mL^{-1}) and an aliquot of the solution was added to the mixture (**PU-1**) at 0°C while stirring, to obtain 2 mol% of catalyst. The mixture (**PU-1-TMG**) was degassed under vacuum and placed in an open mold. The material was cured under vacuum at 0°C and then at room temperature overnight.

PU-1-TPB: TMG film: TPB:TMG was dissolved in anhydrous acetonitrile (ACN) (4.35 mg mL^{-1}) and an aliquot of the solution was added to the mixture (**PU-1**) to obtain 2 mol% of the TPB:TMG. The mixture (**PU-1-TPB:TMG**) was degassed under vacuum and placed in an open mold. The material was cured under vacuum at room temperature and then at 40-50 ^{o}C overnight.

*Synthesis of the cross-linked polyurethane film (**PU-2**)*

Pristine **PU-2** film::Bis(4-hydroxyphenyl) Disulfide (450.6 mg, 1.8 mmol) previously dissolved in 0.2 mL of anhydrous THF was mixed with the tris-isocyanate terminated pre-polymer (1.865 g, 1.2 mmol) and stirred vigorously. The mixture (**PU-2**) was degassed under vacuum and placed in an open mold. The material was cured under vacuum at room temperature and then at 40-50 ^{o}C overnight.

PU-2-TMG film: TMG was dissolved in anhydrous THF (27.6 mg mL^{-1}) and an aliquot of the solution was added to the mixture (**PU-2**) at 0 ^{o}C while stirring, to obtain 2 mol% of catalyst. The mixture (**PU-2-TMG**) was degassed under vacuum and placed in an open mold. The material was cured under vacuum at 0 ^{o}C and then at room temperature overnight.

PU-2-TPB:TMG film: TPB:TMG was dissolved in anhydrous acetonitrile (ACN) (10.5 mg mL^{-1}) and an aliquot of the solution was added to the mixture (**PU-2**) to obtain 2 mol% of the TPB:TMG. The mixture (**PU-2-TPB:TMG**) was degassed under vacuum and placed in an open mold. The material was cured under vacuum at room temperature and then at 40-50 ^{o}C overnight.

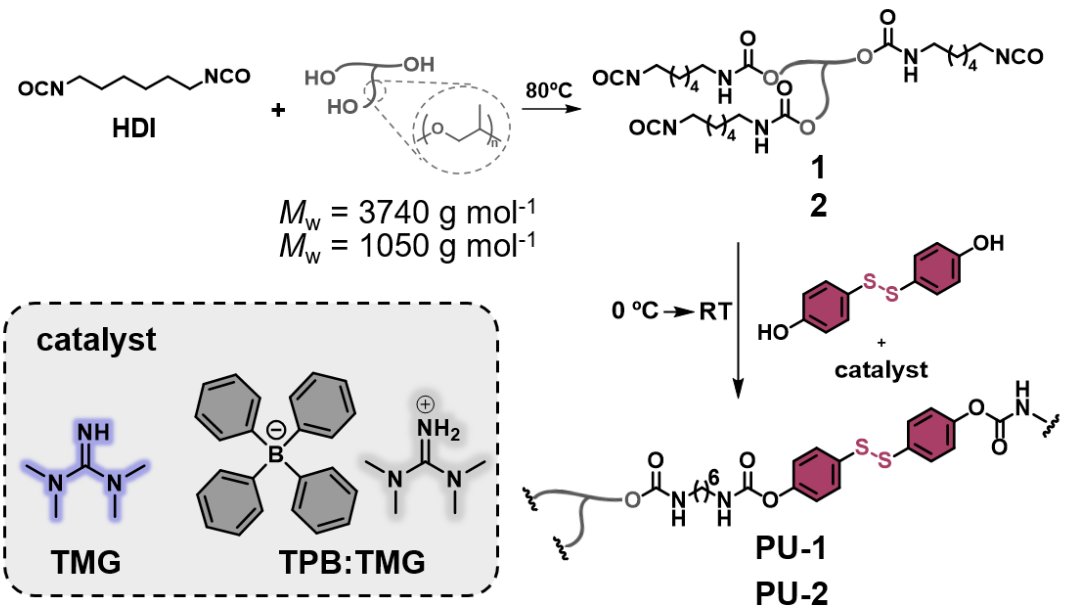

Scheme S1: Synthesis of PU-disulfide networks.

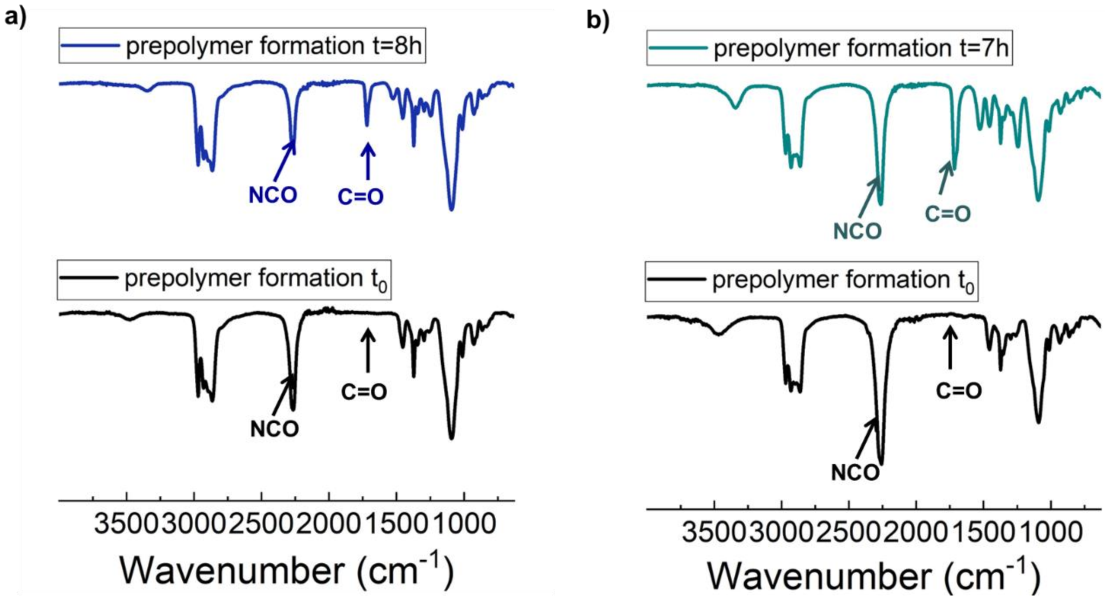

Figure S1: FTIR characterization of a) prepolymer 1: t=0 s and t= 8h and b) prepolymer 2: t = 0 s and t = 7h. The decrease of the characteristic NCO band and the appearance of the urethane band can be observed

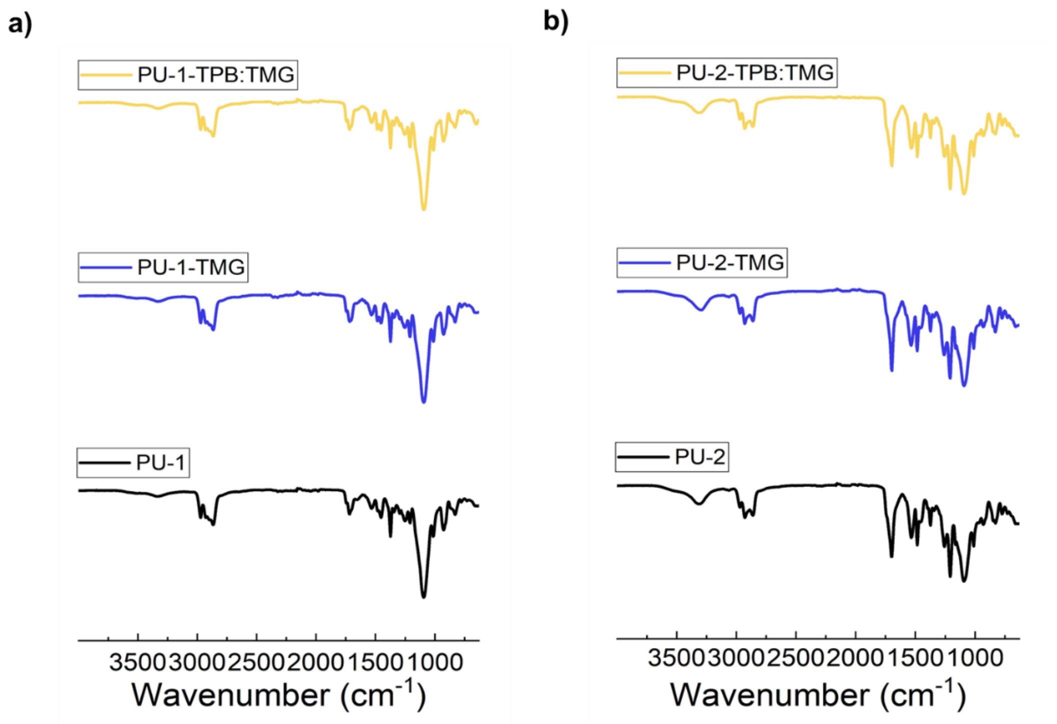

Figure S2. FTIR characterization of PU-1 and PU-2 series of materials. a) FTIR characterization of the crosslinked PU-1 (PU-1, PU-1-TMG and PU-1-TPB:TMG). b) FTIR characterization of the crosslinked PU-2 (PU-2, PU-2-TMG and PU-2-TPB:TMG).

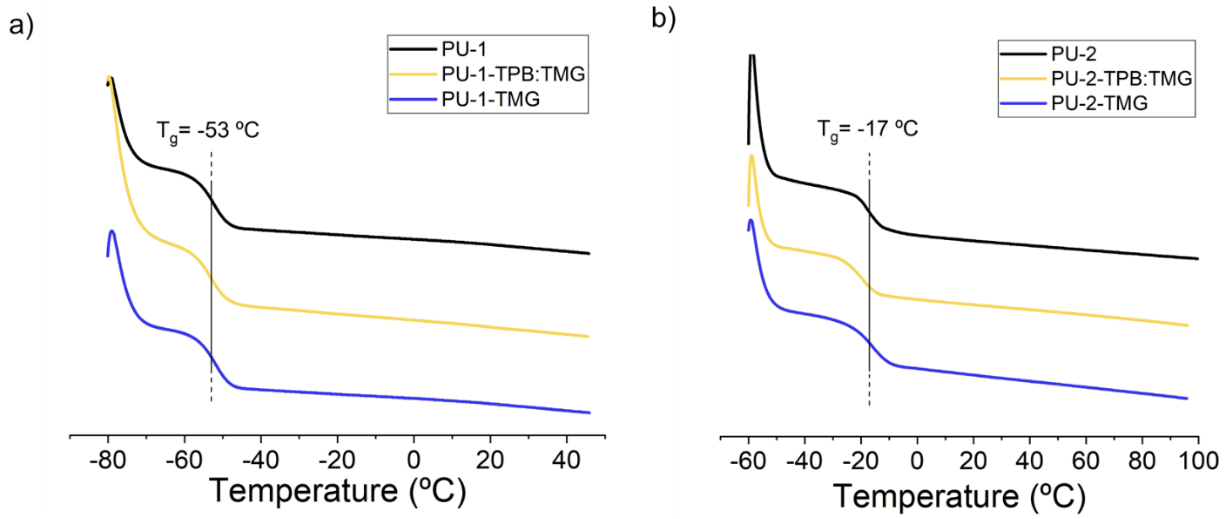

Figure S3. DSC of PU-1 and PU-2 series of materials. a) DSC measurements od PU-1 (pristine), PU-1-TMG and of PU-1-TPB:TMG. b) DSC measurements od PU-2 (pristine), PU-2-TMG and of PU-2-TPB:TMG

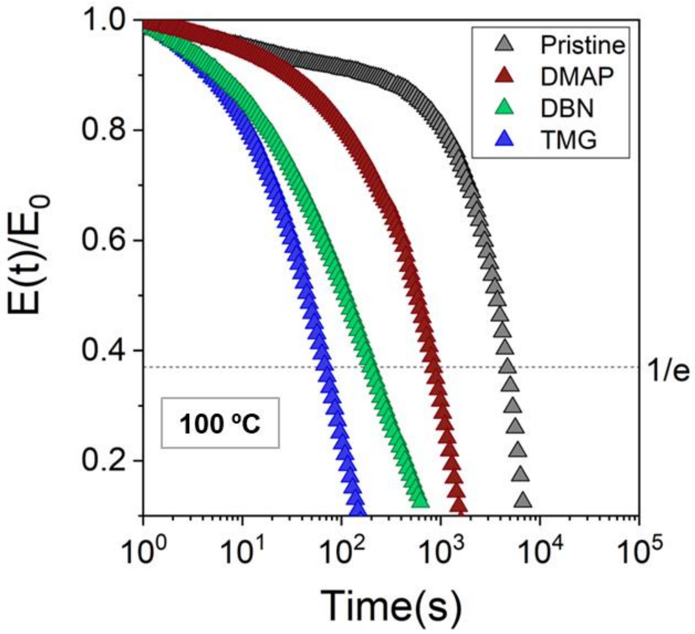

Figure S4: Stress relaxation measurements at 100ºC of PU-1 films containing the 2% mol content of different base catalysts

Model reaction to assess PU exchange

Model reactions were performed using 1,2-di-p-tolyldisulfane and 1,1'-(disulfanediylbis(4,1-phenylene))bis(3-phenylurea) (Figure S1, yellow and black, respectively) in the presence of organic base to confirm that the exchange mechanism was mediated by the base catalyst and not by the presence of any free thiol or amine present in the media. The disulfide exchange was monitored by ^1H NMR spectroscopy and the spectrum was recorded at different reaction times. At time 0 s, the characteristic signals of the symmetric tolyldisulfide (yellow) and urea (black and orange) appear as singlets. After the addition of the catalyst, TMG in 2 mol%, at RT in t < 5 min of reaction, we observed the immediate formation of the statistical products corresponding to the asymmetric disulfide (purple and red signals).

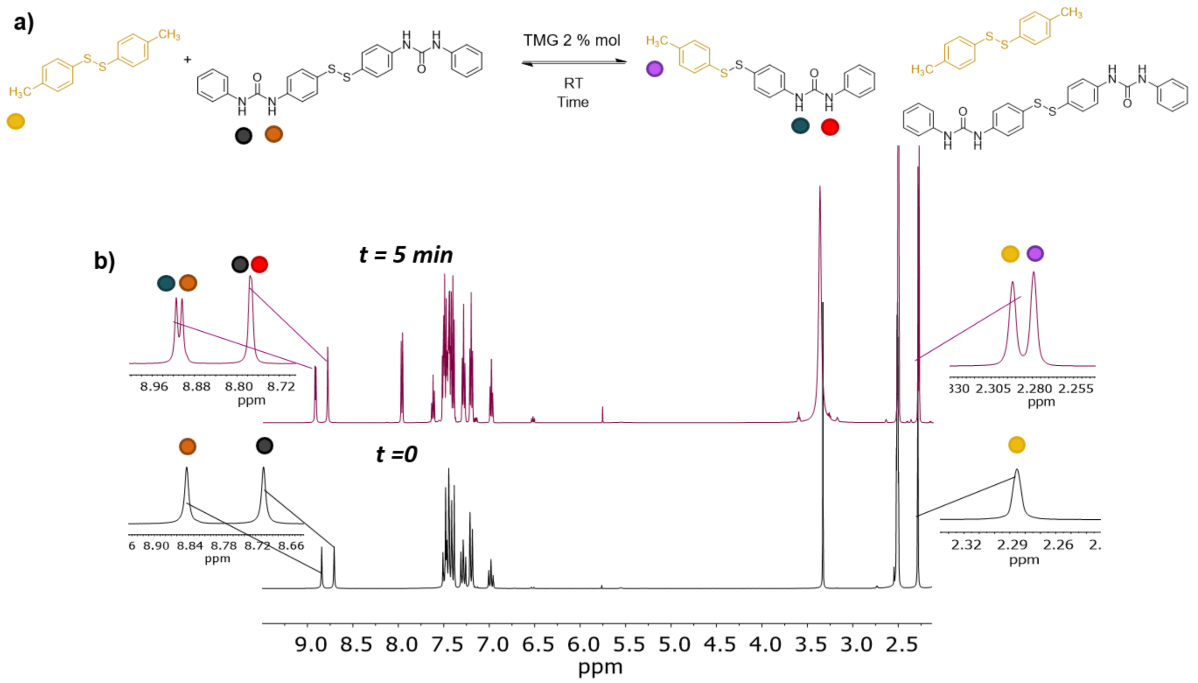

Figure S5: Model reaction showing the base (TMG) catalyzed disulfide exchange. a) scheme of the reaction. b) ^1H NMR of the reaction at t=0 and t= 5 min.

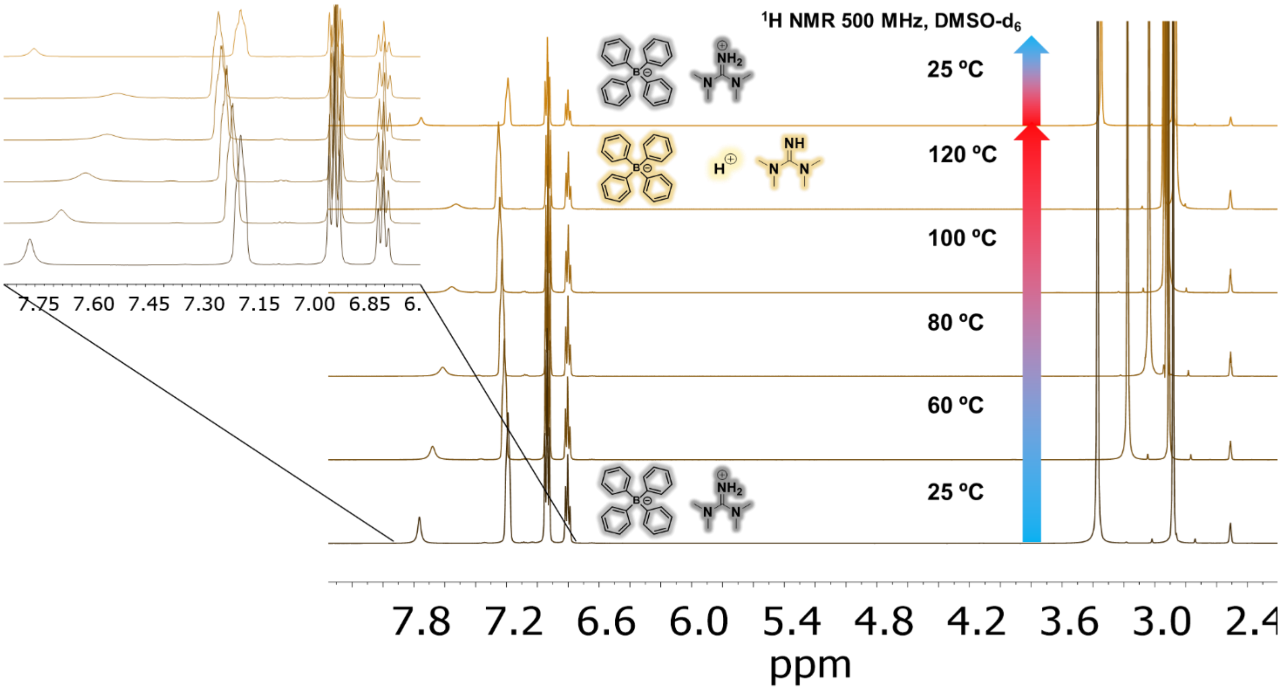

Figure S6: ^{1}H NMR of the thermally-reversible organocatalyst (TPB:TMG), recorded at different temperatures, from 25 °C to 120 °C, and then recorded again at 25 °C after heating up to 120 °C.

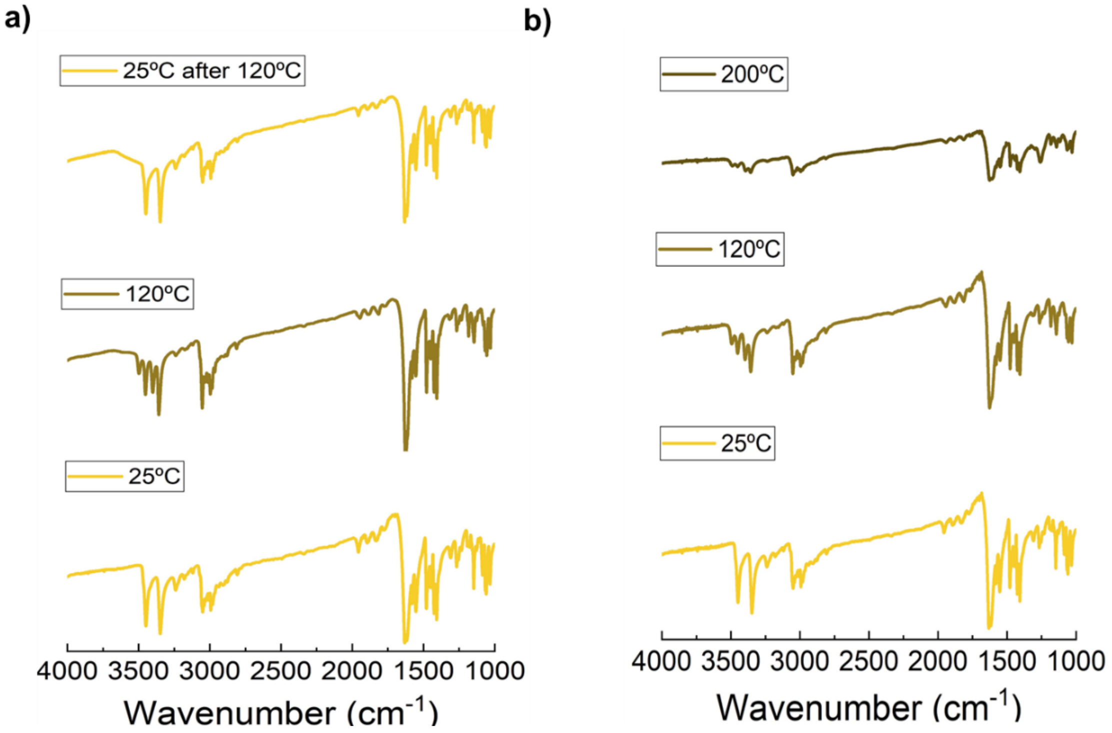

Figure S7: FTIR spectroscopy of the thermally-reversible organocatalyst (TPB: TMG), recorded at different temperatures. a) FTIR analysis conducted at 25 °C,120 °C and again at 25 °C after heating up to 120 °C. b) FTIR analysis conducted at 25 °C,120 °C and 200 °C.

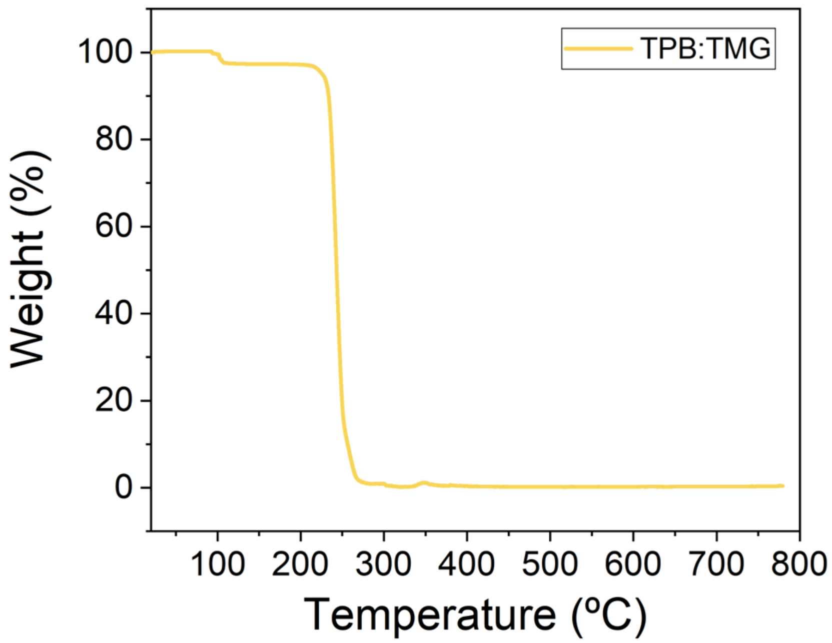

Figure S8: TGA measurement of the thermally-reversible organocatalyst (TPB:TMG)

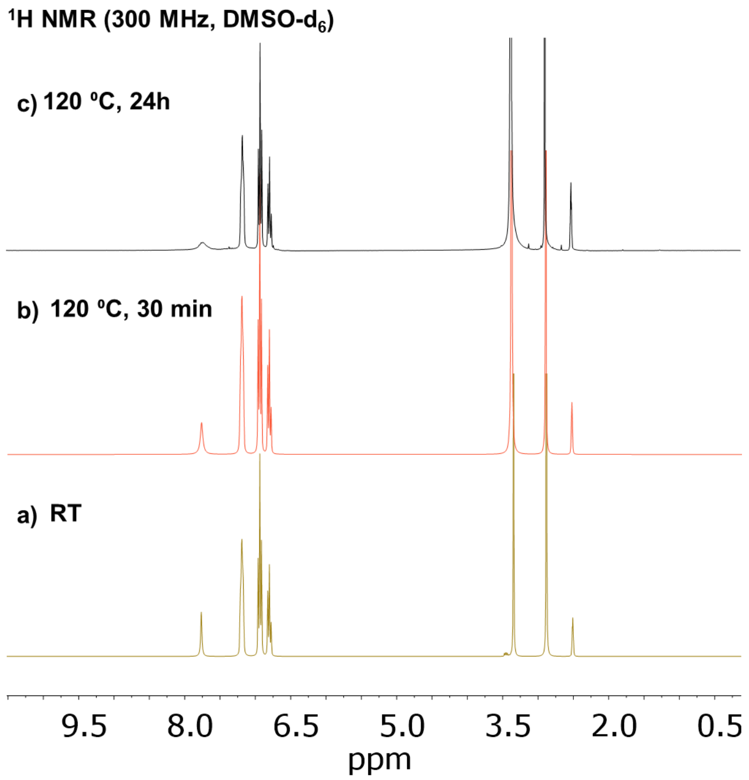

Figure S9: ^{1}H NMR of the thermally-reversible organocatalyst (TPB:TMG). a) ^{1}H NMR recorded at room temperature. b) ^{1}H NMR recorded at room temperature after 30 minutes at 120°C. c) ^{1}H NMR recorded at room temperature. b) ^{1}H NMR recorded at room temperature after 24 hours at 120°C.

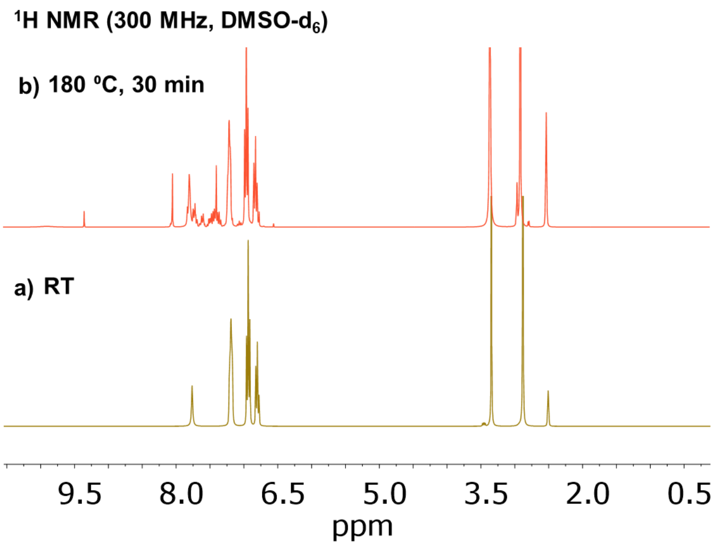

Figure S10: ^{1}H NMR of the thermally-reversible organocatalyst (TPB:TMG). a) ^{1}H NMR recorded at room temperature. b) ^{1}H NMR recorded at room temperature after 30 minutes at 180°C.

Table S1. Data of the E_a and relative error for PU-1 and PU-2 series

Polymer	Additive	E_a (kJ·mol^{-1})*
**PU-1**	**-**	148±14
	**TMG**	95±5
	**TPB:TMG**	179±8 (80 °C ≤ T≤ 100 °C)
		124±4 (100 °C ≤ T≤ 120 °C)
**PU-2**	**-**	126±13
	**TMG**	89±11
	**TPB:TMG**	148±9 (80 °C ≤ T≤ 120 °C)
		87±5 (120 °C ≤ T≤ 140 °C)

*Activation energy obtained from Arrhenius plots

Creep data discussion

The strain curves of the investigated materials exhibit three distinct stages in the creep behavior. Initially, an instantaneous and time-independent elastic deformation is observed, indicating the immediate response of the material to the applied load. Subsequently, a viscoelastic deformation occurs, characterized by a decrease in creep rate over time. Finally, a steady-state deformation rate is reached, and the function ***t σ/ γ(t)*** is constant and corresponds to the Newtonian viscosity. When the load is removed, the materials show an immediate recovery corresponding to the elastic deformation, while the viscoelastic deformation gradually approaches a plateau over time. This behavior can be modeled using the well-known Burgers model.[2] The Burgers model is the one here applied.

$$\gamma(t) = \frac{\sigma_0}{G_1} + \frac{\sigma_0}{G_2}\left(1 - e^{-t/\tau_2}\right) + \frac{\sigma_0}{\eta_3}t$$

In which, G_1 is the modulus of the elastic element, $\tau_2 = \frac{G_2}{\eta_2}$ is the relaxation time of the viscoelastic part, and η_3 is the Newtonian viscosity of the steady –state viscous part.

The strain recovery curve provides easily valuable insights into important viscoelastic parameters, including the maximum compliance, J_{max}, which accounts for the strain achieved prior to load removal, the

nonrecoverable compliance, or final strain, which accounts for the viscous flow during creep experiment, and the instantaneous elastic compliance, J_e^0 which account for the recovered or elastic strain as J_e^0 =J_{max}-J_{nr} If the viscoelastic material is permanently cross-linked, such as in thermosetting polymers, the deformation is purely elastic and corresponds to the equilibrium compliance of the network, that is maintained until the load is removed, as the nonrecoverable strain is negligible. Conversely, for vitrimers, the temperature controls the dynamic bonds: depending on their activation, the deformation can be predominately viscous or elastic.

Table S2 and **Table S3** represent a concise summary of significant data.

Table S2. Summary of significant data of PU-1 series: the parameters J_e^0 and J_{nr} determined through the creep and recovery test, and η_3, obtained by applying the Burger model

	T (ºC)	J_e^0	J_{nr}	η_3*
**PU-1**	**60**	$1.69{\cdot}10^{-6}$	$2.00{\cdot}10^{-8}$	$4.00{\cdot}10^{11}$
	**100**	$1.53{\cdot}10^{-6}$	$4.92{\cdot}10^{-8}$	$8.00{\cdot}10^{10}$
	**120**	$1.49{\cdot}10^{-6}$	$4.8{\cdot}10^{-8}$	$1.09{\cdot}10^{10}$
**PU-1-TMG**	**60**	$1.65{\cdot}10^{-6}$	$3.46{\cdot}10^{-8}$	$8.00{\cdot}10^{9}$
	**100**	$2.6{\cdot}10^{-6}$	$9.40{\cdot}10^{-7}$	$3.09{\cdot}10^{9}$
	**120**	$5.42{\cdot}10^{-6}$	$9.90{\cdot}10^{-6}$	$4.00{\cdot}10^{7}$
**PU-1-TPB:TMG**	**60**	$7.19{\cdot}10^{-7}$	$2.46{\cdot}10^{-8}$	$8.0{\cdot}10^{10}$
	**100**	$1.57{\cdot}10^{-6}$	$3.90{\cdot}10^{-8}$	$8.0{\cdot}10^{9}$
	**120**	$2.3{\cdot}10^{-6}$	$9.9{\cdot}10^{-7}$	$4.0{\cdot}10^{9}$

*Data obtained by applying the Burgers model

Table S3. Summary of significant data of PU-2 series: the parameters J_e^0 and J_{nr} determined through the creep and recovery test, and η3, obtained by applying the Burger model

	T (ºC)	J_e^0	J_{nr}	η_3*
**PU-2**	**60**	$6.90{\cdot}10^{-7}$	$3.78{\cdot}10^{-8}$	$4.00{\cdot}10^{11}$
	**80**	$5.05{\cdot}10^{-7}$	$3.40{\cdot}10^{-8}$	-
	**100**	$3.27{\cdot}10^{-7}$	$1.10{\cdot}10^{-7}$	$8.00{\cdot}10^{10}$
	**120**	$4.05{\cdot}10^{-7}$	$1.20{\cdot}10^{-7}$	$4.00{\cdot}10^{10}$
**PU-2-TMG**	**60**	$1.00{\cdot}10^{-6}$	$1.38{\cdot}10^{-7}$	$2.22{\cdot}10^{10}$
	**80**	$1.55{\cdot}10^{-6}$	$5.28{\cdot}10^{-7}$	$4.05{\cdot}10^{9}$
	**100**	$1.48{\cdot}10^{-6}$	$6.52{\cdot}10^{-7}$	$8.05{\cdot}10^{9}$
	**120**	$1.52{\cdot}10^{-6}$	$7.76{\cdot}10^{-7}$	$3.55{\cdot}10^{8}$
**PU-2-TPB:TMG**	**60**	$4.05{\cdot}10^{-7}$	$2.88{\cdot}10^{-8}$	$7.0{\cdot}10^{10}$
	**80**	$3.27{\cdot}10^{-7}$	$2.14{\cdot}10^{-8}$	$9.0{\cdot}10^{10}$
	**100**	$5.05{\cdot}10^{-7}$	$1.50{\cdot}10^{-7}$	$9.56{\cdot}10^{9}$
	**120**	$6.90{\cdot}10^{-7}$	$3.38{\cdot}10^{-7}$	$9.80{\cdot}10^{9}$

*Data obtained by applying the Burgers model

Figures S11 and S12 depict the non-recoverable compliance (J_{nr}) values with temperature of PU-1 and PU-2 material series. J_{nr} refers to the component of the total compliance that is not recoverable after the removal of an applied load. The non-recoverable compliance quantifies the irreversible or viscous deformation of a material. In other words, it measures the residual deformation that is not recovered upon stress release. The non-recoverable compliance is an important parameter in characterizing the viscoelastic behavior of materials, providing the extent of irreversible deformation in polymers.

Through a comparative analysis of J_{nr} values at different temperatures for PU (pristine), PU-TMG, and PU-TPB:TMG (figures S11 and S12), interesting trends emerge. it is evident that the material containing TMG as permanently activated catalyst exhibits the highest J_{nr} values, whereas the pristine materials show a predominantly elastic-like behaviour (lower J_{nr}) Interestingly, the PU-TPB:TMG materials exhibit a temperature-dependent behavior; initially, at temperatures below 100 ^{o}C, these materials resemble more the pristine samples (lower J_{nr} values). However, as the temperature rises to 120 ^{o}C, the PU-TPB:TMG approach to the PU-TMG values of J_{nr}, indicating the materials exhibit a more pronounced irreversible deformation.

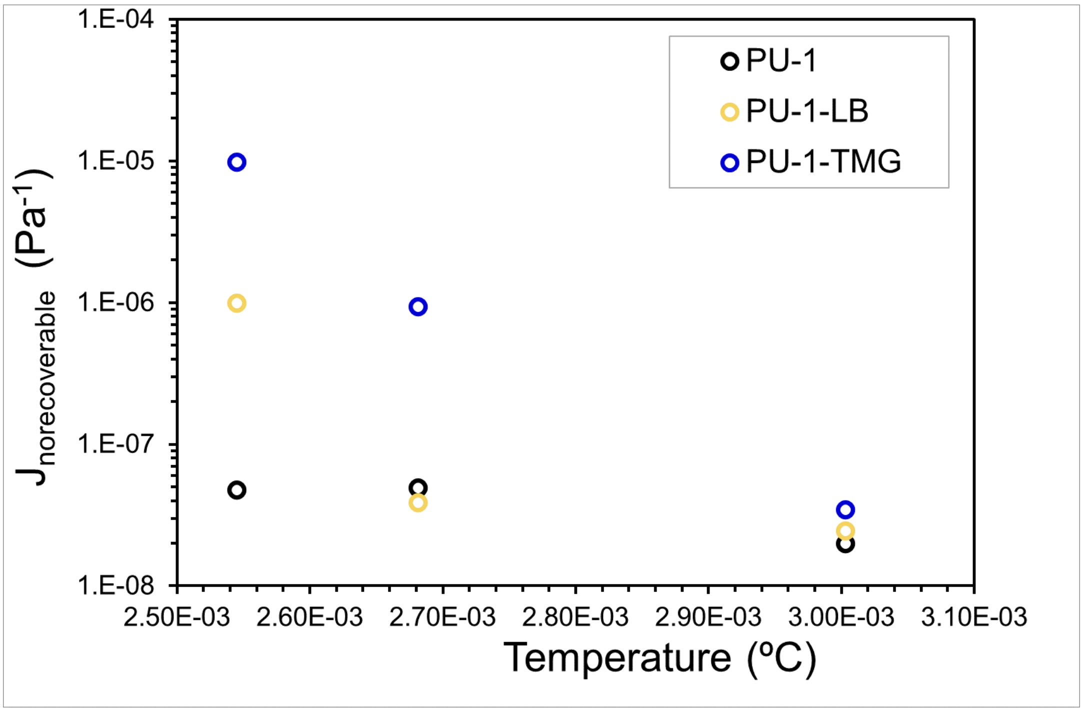

Figure S11: Representation of Jnr with temperature of PU-1 series

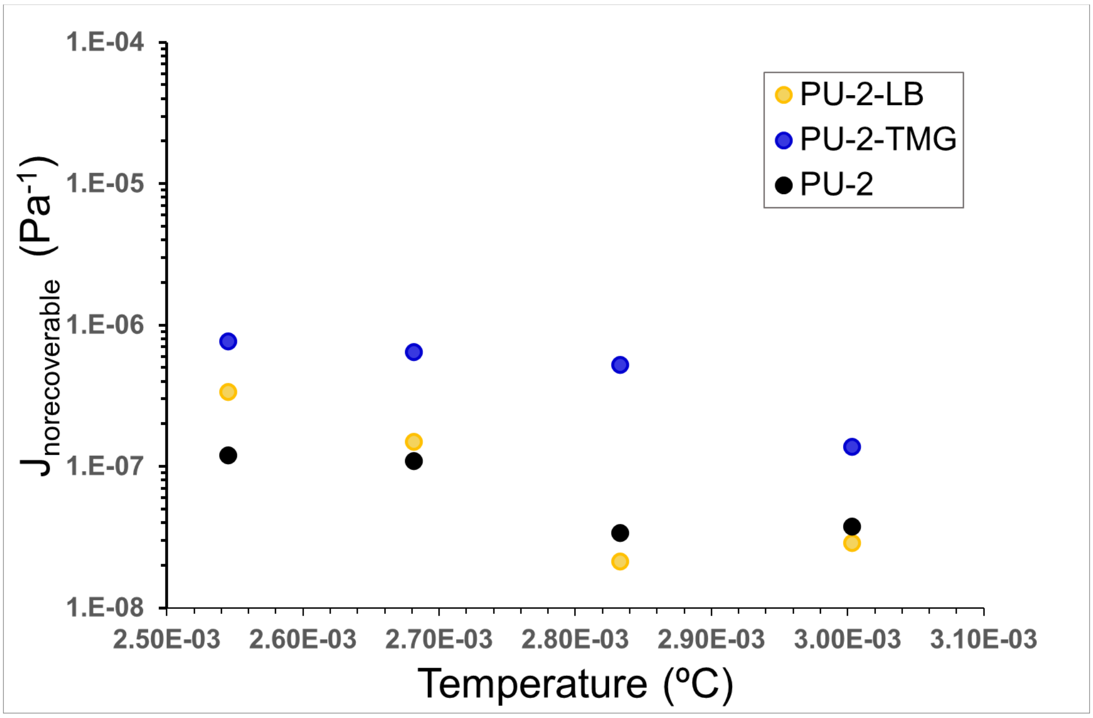

Figure S12: Representation of Jnr with temperature of PU-2 series

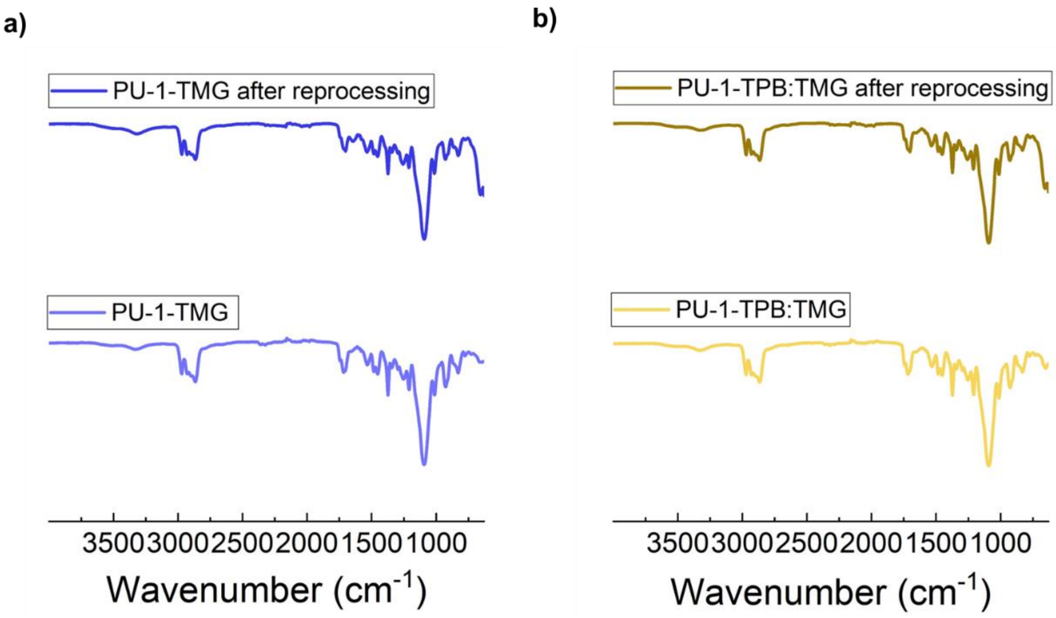

Figure S13: FTIR characterization of the crosslinked PU-1 networks. a) FTIR of PU-1-TMG before and after reprocessing. b) FTIR of PU-1-TPB:TMG, before and after reprocessing

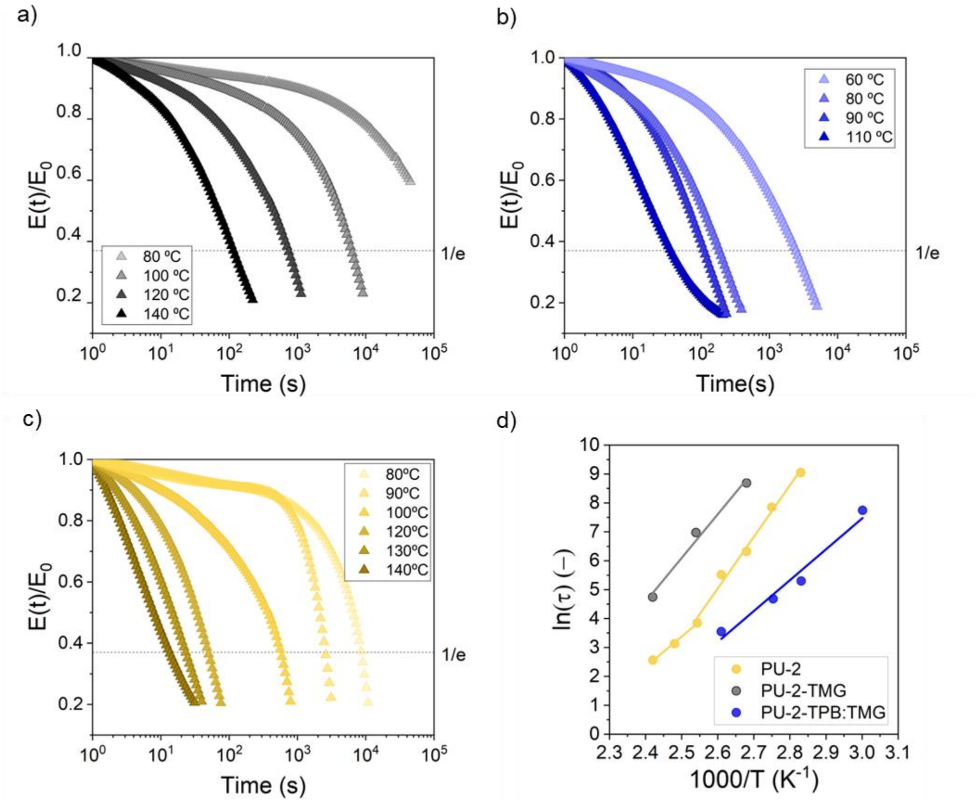

Figure S14: Stress relaxation measurements and Arrhenius plot of PU-2 series. a) Stress relaxation at different temperatures of PU-2 (pristine). b)) Stress relaxation at different temperatures of PU-2-TMG. c)) Stress relaxation at different temperatures of PU-2-TPB:TMG. d) Arrhenius plot of PU-2 series

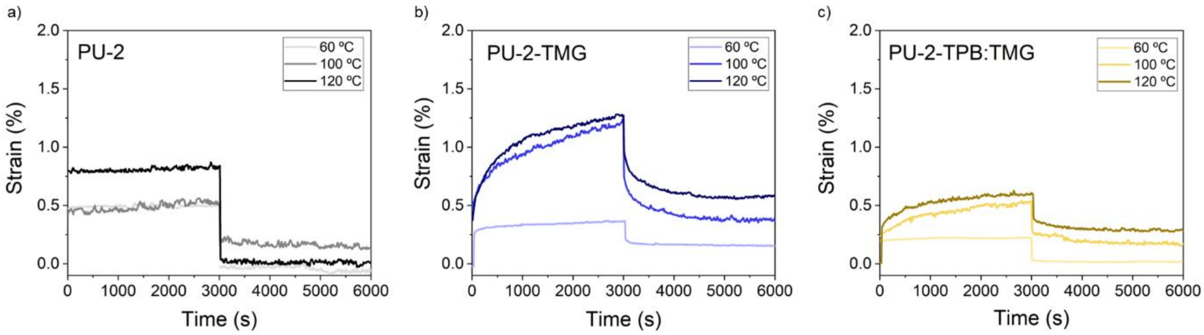

Figure S15: Creep experiments on PU-2 series. a) Creep measurements of PU-2 (pristine) at 60 °C, 100 °C, and 120 °C. b) Creep measurements of PU-2-TMG at 60 °C, 100 °C, and 120 °C. c) Creep measurements of PU-2-TPB:TMG at 60 °C, 100 °C, and 120 °C

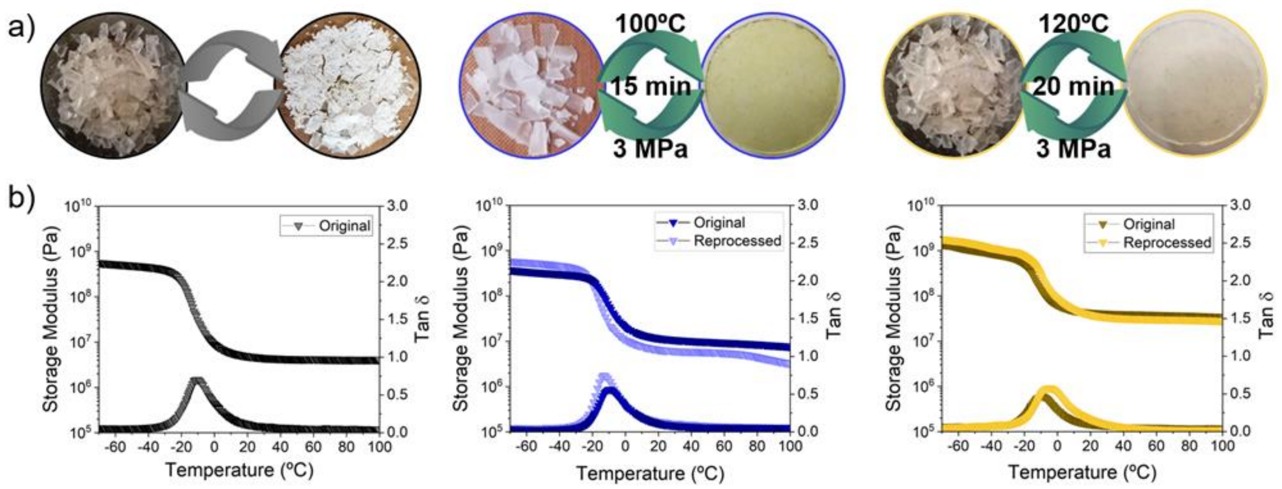

Figure S16. Reprocessing conditions and Dynamic mechanical analysis (DMA) of PU-2 series. a) Reprocessing conditions of PU-2, PU-TMG, and PU-TPB:TMG materials. b) DMA experiments of PU-2 (pristine) before reprocessing, of PU-2-TMG and comparison with the reprocessed sample, and of PU-2-TPB:TMG and comparison with the reprocessed sample.

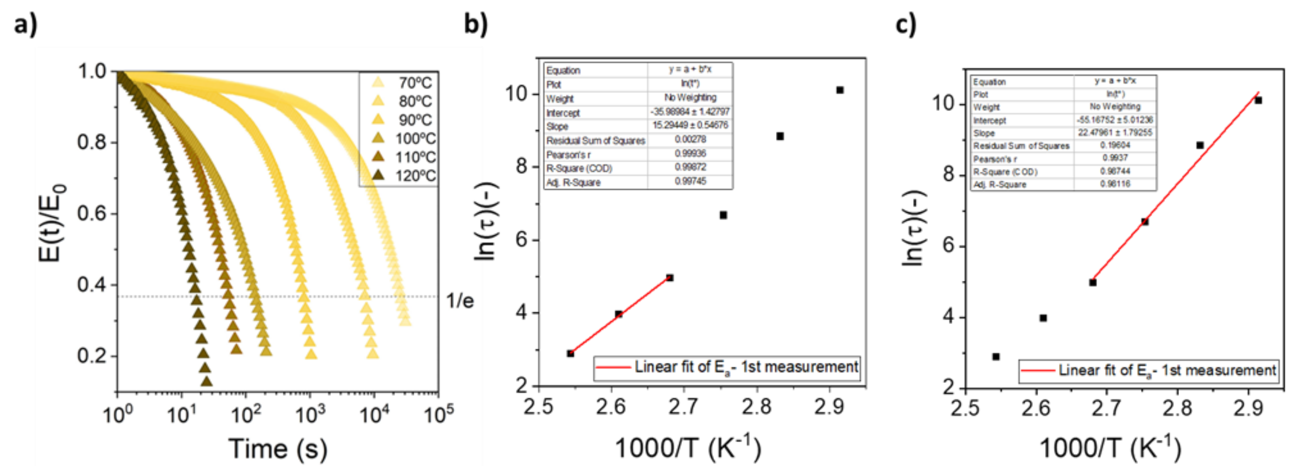

Figure S17: Stress relaxation and Arrhenius plot curves for of the PU-1 series (1st measurement)

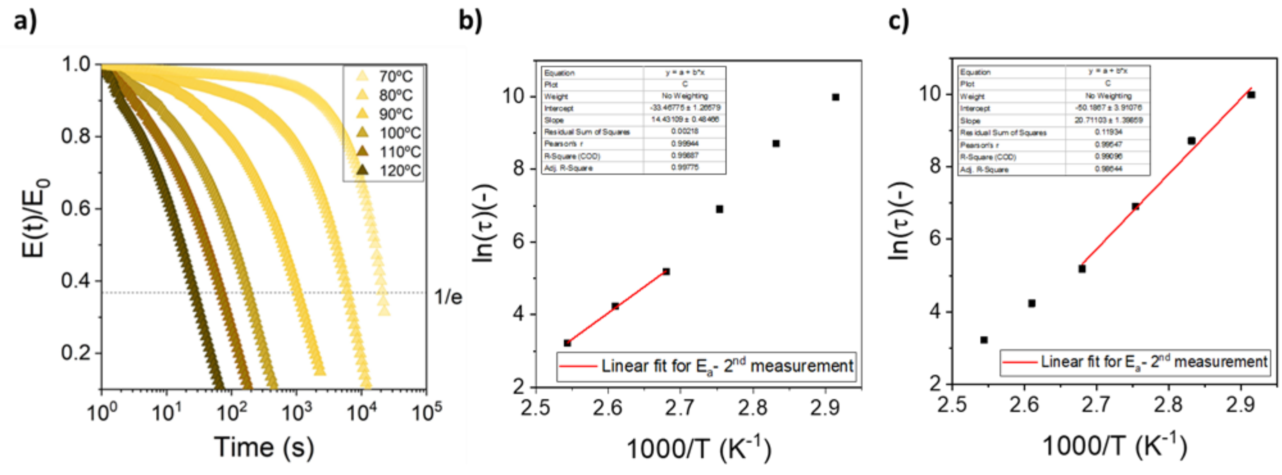

Figure S18: Stress relaxation and Arrhenius plot curves for of the PU-1 series (2nd measurement)

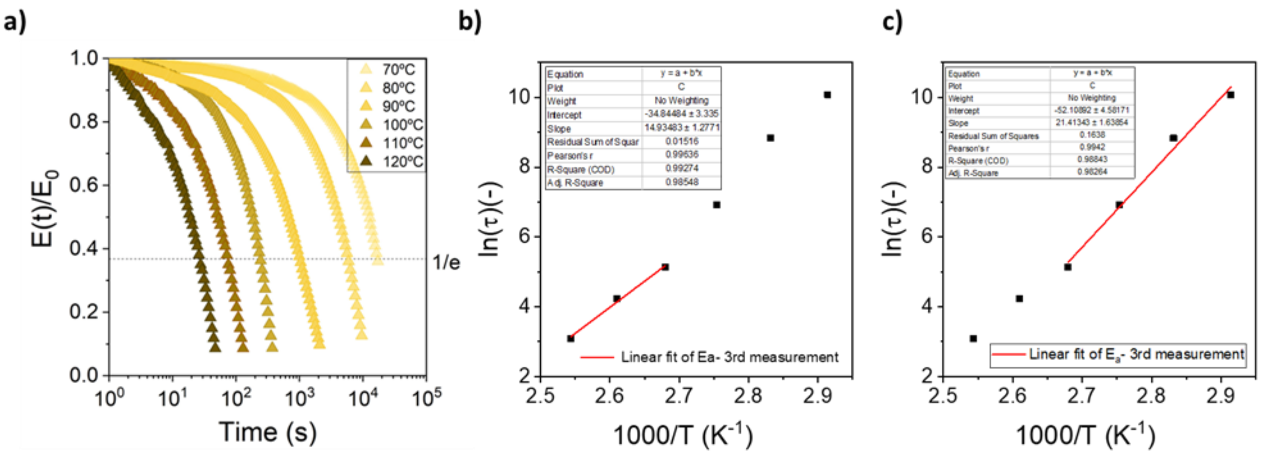

Figure S19: Stress relaxation and Arrhenius plot curves for of the PU-1 series (3rd measurement)

Table S4. Data of the E_a and relative error for PU-1 (the three measurements for PU-1-TPB:TMG are shown)

Polymer	Additive	E_a ($kJ\cdot mol^{-1}$)*
**PU-1**	**-**	148±14
	**TMG**	95±5
	**TPB:TMG (1st meas.)**	127±5 (80 ºC ≤ T≤ 100 ºC)
		187±15 (100 ºC ≤ T≤ 120 ºC)
	**TPB:TMG (2nd meas.)**	120±4 (80 ºC ≤ T≤ 100 ºC)
		172±11 (100 ºC ≤ T≤ 120 ºC)
	**TPB:TMG (3rd meas.)**	124±11(80 ºC ≤ T≤ 100 ºC)
		178±14 (100 ºC ≤ T≤ 120 ºC)

Bibliography

1 F. Gamardella, S. Muñoz, S. De la Flor, X. Ramis and A. Serra, *Polymers*, 2020, **12**, 2913.

2 F. Van Lijsebetten, K. De Bruycker, Y. Spiesschaert, J. M. Winne and F. E. Du Prez, *Angew. Chem. Int. Ed.*, , DOI:10.1002/anie.202113872.
